# Targeted Nanotherapeutics for Respiratory Diseases: Cancer, Fibrosis, and Coronavirus

**DOI:** 10.1002/adtp.202000203

**Published:** 2020-10-13

**Authors:** Joydeb Majumder, Tamara Minko

**Affiliations:** ^1^ Department of Pharmaceutics Ernest Mario School of Pharmacy, Rutgers The State University of New Jersey Piscataway NJ 08854 USA

**Keywords:** coronavirus, cystic fibrosis, lung cancer, nanotherapeutics, targeted delivery

## Abstract

Systemic delivery of therapeutics for treatment of lung diseases has several limitations including poor organ distribution of delivered payload with relatively low accumulation of active substances in the lungs and severe adverse side effects. In contrast, nanocarrier based therapeutics provide a broad range of opportunities due to their ability to encapsulate substances with different aqueous solubility, transport distinct types of cargo, target therapeutics specifically to the deceased organ, cell, or cellular organelle limiting adverse side effects and increasing the efficacy of therapy. Moreover, many nanotherapeutics can be delivered by inhalation locally to the lungs avoiding systemic circulation. In addition, nanoscale based delivery systems can be multifunctional, simultaneously carrying out several tasks including diagnostics, treatment and suppression of cellular resistance to the treatment. Nanoscale delivery systems improve the clinical efficacy of conventional therapeutics allowing new approaches for the treatment of respiratory diseases which are difficult to treat or possess intrinsic or acquired resistance to treatment. The present review summarizes recent advances in the development of nanocarrier based therapeutics for local and targeted delivery of drugs, nucleic acids and imaging agents for diagnostics and treatment of various diseases such as cancer, cystic fibrosis, and coronavirus.

## Graphical abstract

Targeted delivery of nanotherapeutics specifically to diseased lungs, pulmonary cells, and/or specific organelles offers distinct advantages over systemic delivery by enhancing efficacy of the treatment and limiting severe adverse side effects. Nanoscale based systems can transport various types of payload, simultaneously carry out several distinct tasks and perform multiple function in diagnosis, treatment, and suppression of cellular resistance.

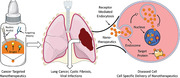

## 1 Introduction

**Figure 1:**
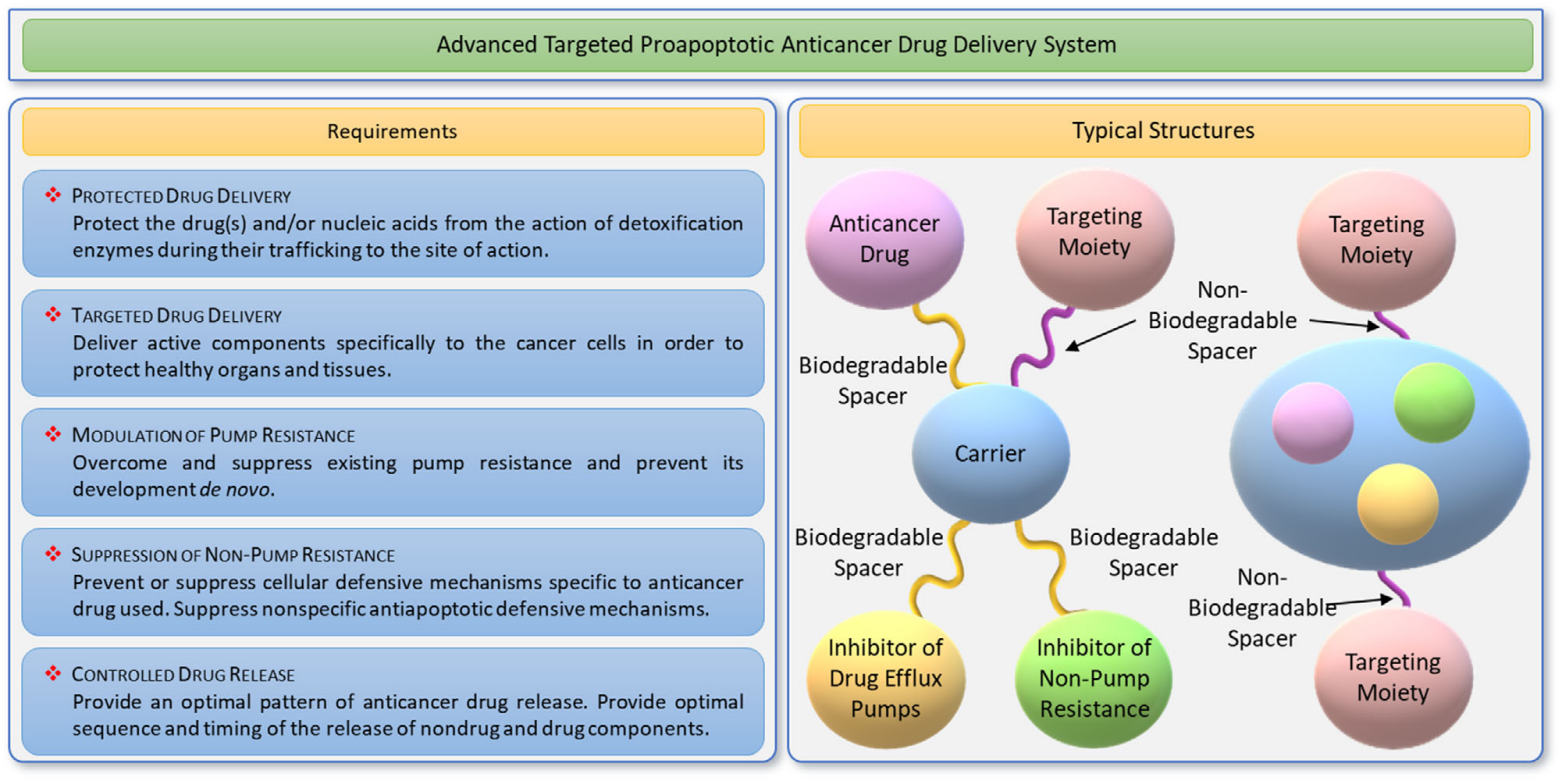
A design principle and typical structures of an advanced targeted proapoptotic drug delivery systems for effective treatment cancers with limiting adverse side effects upon healthy organs, tissues, and cells.

Lung diseases are one of the main causes of death among both men and women worldwide. The mortality rates for lung diseases have been increasing by each year.^[^ ^1^, ^2^ ^]^ Therefore, methods of developing new therapeutic solutions as well as improving the current therapies for the common lung diseases such as asthma, cystic fibrosis, chronic obstructive pulmonary disease, lung cancer, and coronavirus infections remain the main focus in the fields of targeted drug delivery. The widely utilized conventional drug delivery methods usually induce adverse side effects.^[^ ^3^, ^4^ ^]^ Recent development of nanoscale‐based systems opens a door for a better delivery of therapeutics by addressing the limitations of conventional therapy. Nanocarrier‐based drug delivery systems can increase bioavailability of poorly water‐soluble therapeutics and address other barriers and shortcomings of traditional drugs.^[^ ^5^ ^]^ While majority of the marketed drugs are poorly water soluble, which limits their administration at high doses,^[^ ^6^, ^7^ ^]^ nanoscale‐based drug delivery systems were found to improve solubility and increase therapeutic efficacy of free non‐bound drugs.^[^ ^8^, ^9^ ^]^ Similarly, biomacromolecular therapeutics such as nucleic acids (DNA, small interfering RNA, antisense oligonucleotides, etc.) are usually degraded in the biological fluids and difficult to deliver at their target site.^[^ ^10^, ^11^ ^]^ However, delivery of nucleic acids via nanocarrier based systems increased their stability and concentration at the target site.^[^ ^12^, ^13^, ^14^, ^15^, ^16^ ^]^ Moreover, nanoscale drug delivery systems can be administered via different routes, such as intravenous,^[^ ^17^, ^18^ ^]^ oral,^[^ ^19^, ^20^ ^]^ and inhalation^[^ ^15^, ^16^, ^21^, ^22^, ^23^, ^24^, ^25^, ^26^, ^27^, ^28^, ^29^, ^30^ ^]^ routes. Furthermore, nanoscale delivery systems are less toxic and immunogenic than the traditional viral vector‐based gene delivery systems.^[^ ^31^, ^32^, ^33^ ^]^ Because of such handful advantages, researchers around the world have been applied significant efforts in recent years to develop various nanosized carriers for targeted delivery of therapeutics. Over the decade, several nanocarrier‐based therapeutics were also approved by FDA for clinical application.^[^ ^34^, ^35^ ^]^ Thus, the recent development of wide spectrum nanoscale systems introduced a new way in diagnosis, treatment and prevention of diseases. Previously, we formulated main requirements and basic concept of effective drug and nucleic acid delivery systems for effective treatment of diseases including cancer.^[^ ^36^ ^]^ To enhance treatment effectiveness, the advanced system should provide for a 1) protected delivery of active components in order to prevent their degradation during its journey to targeted cells; 2) targeted transport specifically to the site of action with the aim of limiting adverse side effects of treatment upon healthy organs, tissues, and cells; 3) modulation of pump drug resistance with the purpose to prevent drug efflux from the diseased cells; 4) suppression of nonpump resistance in order to overcome other resistance mechanisms non related to drug efflux pumps; and 5) controlled release of active components in a predefined desired manner.^[^ ^36^, ^37^, ^38^, ^39^, ^40^, ^41^, ^42^, ^43^, ^44^, ^45^ ^]^ These main requirements concretized for cancer treatment and general composition of advanced proapoptotic drug delivery system are shown in **Figure** **1**. In this review, we summarize recent reports on the development of various nanotherapeutics including nanocarrier‐based drugs and nucleic acids for the treatment of lung diseases with emphasis on lung cancer, idiopathic pulmonary, and cystic fibrosis and recent coronavirus infections.^[^ ^15^, ^16^, ^23^, ^24^, ^46^, ^47^, ^48^, ^49^, ^50^ ^]^

## 2 Targeted Nanotherapeutics for Lung Diseases: Active and Passive Targeting

**Figure 2:**
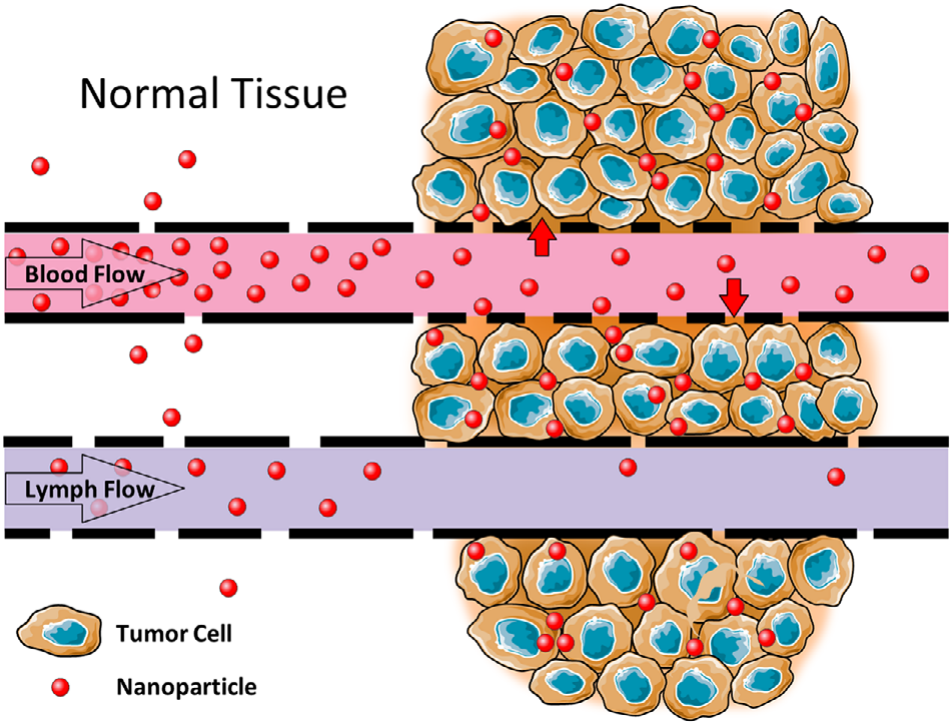
A schematic representation of the Enhanced Permeability and Retention (EPR) effect. The leaky vasculature and dysfunctional lymphatics of tumors allow the preferential accumulation and retention of high molecular weight nanoparticles in solid tumors. Reproduced with permission.^[^ ^51^ ^]^ Copyright 213, Hindawi.

**Figure 3:**
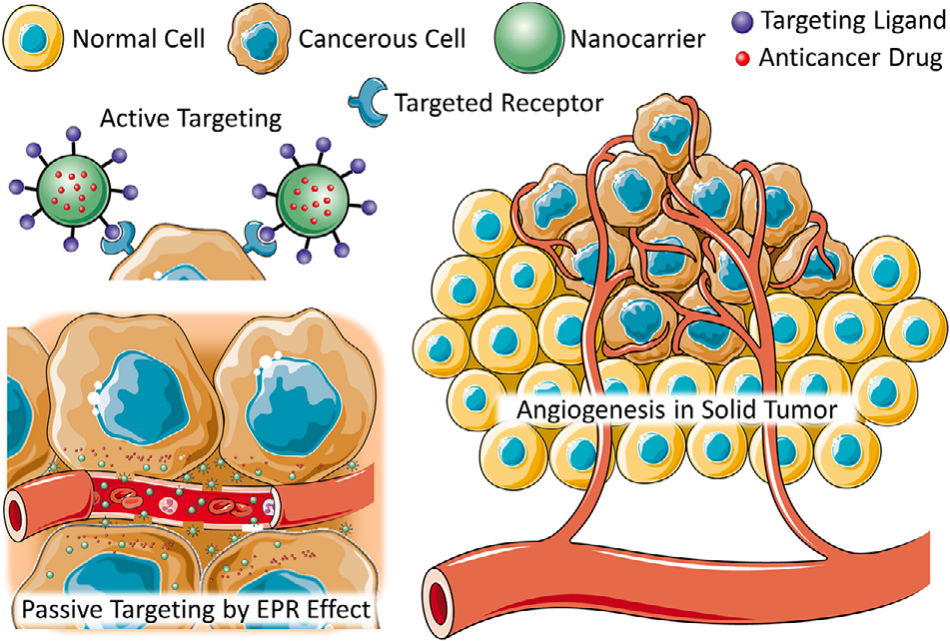
Passive and active targeting cancer cells. Passive targeting depends on the enhance permeability and retention (EPR) effect. Active targeting may be achieved by enabling the uptake of nanotherapeutics by receptor mediated endocytosis. For instance, nanoparticles may be decorated with a ligand targeting receptors (or other molecules) overexpressed on the plasma membrane of cancer cells.

**Figure 4:**
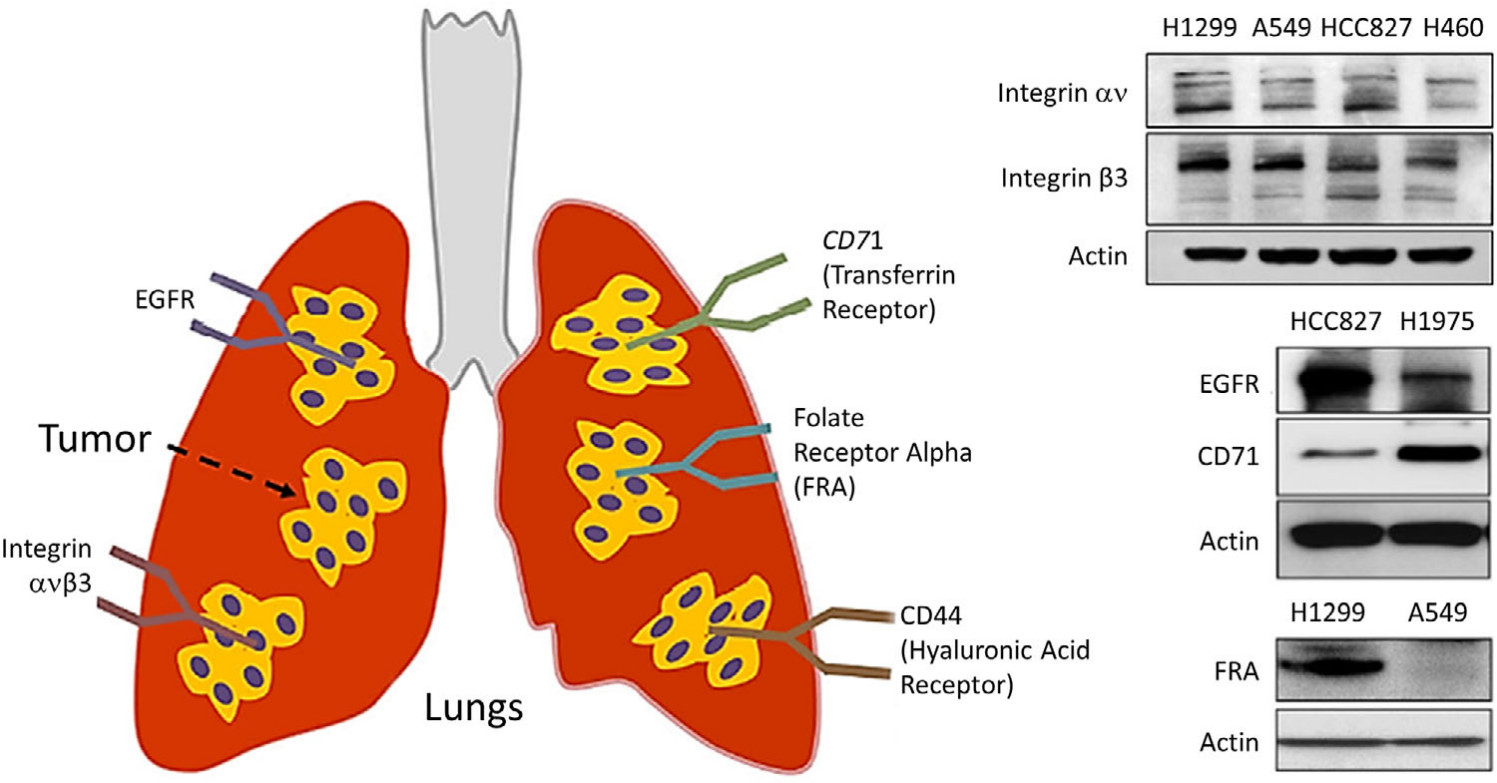
Different plasma membrane receptors overexpressed in lung cancer cells. Western blots on the right panel show the expression of these receptors in different lung cancer cells. Reproduced with permission.^[^ ^48^ ^]^ Copyright 2017, Springer.

**Figure 5:**
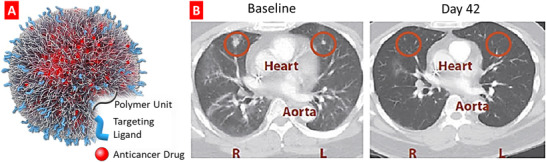
The impact of the nanotherapeutic BIND‐014, a polymeric nanodrug carrier, encapsulating docetaxel, and targeting prostate specific membrane antigen on human drug metastases from a primary cholangiocarcinoma. A) A schematic representation of BIND‐014 composed of a biodegradable and hydrophobic PLA polymeric core and a hydrophilic PEG corona decorated with small‐molecule targeting ligands, and an encapsulated anticancer drug (docetaxel). Adapted with permission.^[^ ^82^ ^]^ Copyright 2013, Dovepress. B) Representative axial images from contrast‐enhanced CT scans obtained from a patient with lung metastases at baseline and at day 42 after two treatment cycles of BIND‐014. Red circles indicate locations of metastatic lesions observed in the baseline CT scan. Adapted with permission.^[^ ^83^ ^]^ Copyright 2012, American Association for the Advancement of Science.

Targeted drug delivery of therapeutics is aimed at transporting of the administered active component predominately at a desired site of action limiting its accumulation in healthy organs and tissues. This goal can be achieved by both passive and active targeting of drugs.^[^ ^37^, ^52^, ^53^, ^54^ ^]^ In passive targeting, high molecular weight substances are accumulated in targeted cells because of specific pathophysiological characteristics of the diseased cells and surrounding microenvironment. For instance, passive targeting to solid tumors is dependent on the enhanced permeability of vessels that supply blood, oxygen, and nutrients to the tumor and limited lymphatic drainage from the tumor environment. This phenomenon was termed as the enhanced permeability and retention (EPR) effect.^[^ ^55^ ^]^ A schematic representation of the EPR effect is displayed in **Figure** **2**. However, the efficiency of passive targeting is limited and the conditions responsible for the EPR effect is not attributed for all diseased tissues which often develop specific mechanisms for resisting traditional treatment approaches.^[^ ^56^ ^]^ On the other hand, active targeting is achieved mainly by decorating the surface of the nanocarriers with targeting moieties such as antibodies,^[^ ^57^ ^]^ proteins,^[^ ^58^ ^]^ peptides,^[^ ^59^ ^]^ aptamers,^[^ ^60^, ^61^, ^62^ ^]^ lectins, carbohydrates, and glycoproteins,^[^ ^63^ ^]^ small molecules,^[^ ^64^, ^65^, ^66^, ^67^, ^68^ ^]^ etc. which have strong affinity to their cellular binding partners such as tumor antigens, cell surface receptors, tumor vasculature.^[^ ^69^ ^]^ Such active targeting nanocarriers are designed to increase the accumulation of therapeutics at their site of actions with limiting exposure to other healthy organs thereby reducing the risk of adverse side effects.^[^ ^70^, ^71^, ^72^, ^73^, ^74^, ^75^ ^]^ Nanocarrier‐based passive and active targeting of cancerous cells and their advantages are presented in **Figure** **3**. Several receptor proteins (such as receptors specific for folate, LHRH, transferrin, etc.) are often overexpressed in tumor cells. A schematic demonstration of various overexpressed cell surface receptors in lung under pathological conditions is outlined in **Figure** **4**. Western blot analysis revealed the presence of the targeting receptor proteins such as folate receptor alpha (FRA), epidermal growth factor receptor (EGFR), integrin etc. in different lung cancer cells as shown in the right panel of Figure 4. Folic acid, transferrin etc. were widely explored as affinity ligands for targeting many tumors cells.^[^ ^76^, ^77^, ^78^ ^]^ For example, recently, researcher developed a gold nanocarrier loaded with drug Aurimmune CYT‐6091 and functionalized with tumor necrosis factor alpha (TNF‐α) using polyethylene glycol (PEG) linker for treating lung cancer. TNF‐α was used as both targeting and therapeutic agent.^[^ ^79^ ^]^ Bind‐014 is another nanocarrier based therapeutics which was investigated in phase II clinical trial for patients with non‐small cell lung cancer.^[^ ^80^ ^]^ Bind‐014 is a polylactic acid (PLA) based nanocarrier system wherein the anticancer drug docetaxel was entrapped. The surface of this system was coated with PEG and targeting ligands against prostate‐specific membrane antigen (PSMA) which is usually abundant in prostate cancer cells as well as in the non‐prostate cancers, such as NSCLC.^[^ ^81^ ^]^ **Figure** **5**A represented a schematic illustration of BIND‐014 composed of a hydrophobic PLA polymeric core and a hydrophilic PEG corona decorated with small‐molecule targeting ligands, and an encapsulated anticancer drug docetaxel.^[^ ^82^ ^]^ The CT scans acquired from a patient suffering from a primary cholangiocarcinoma revealed regression of the lung metastases after two cycles of BIND‐014 treatment (Figure 5B).^[^ ^83^ ^]^ These results indicated that Bind‐014 was clinically active and nontoxic for NSCLC.

## 3 Nanotherapeutics for Lung Diseases

### 3.1 Lung Cancer

Lung cancer is the prime cause of cancer death worldwide. Chemotherapy, radiation therapy and their combination with surgery are the current therapeutic options for all type of lung cancers.^[^ ^84^ ^]^ In most cases of lung cancer chemotherapy, drugs are often administered intravenously, and they can circulate throughout the body affecting both normal and cancer cells. Over the last two decades, various nanoparticles (such as metal‐based nanoparticles, lipid‐based nanoparticles, polymeric nanoparticles, etc.) were explored for targeted therapeutic delivery and diagnostic applications (or combination of both in one theranostic system).^[^ ^51^, ^85^, ^86^ ^]^ However, the translational application of naked metallic nanoparticles (as imaging contrast agents) is limited due to their toxicity.^[^ ^87^, ^88^ ^]^ Therefore, lipid and polymer‐based nanoparticles have received more attention of the researchers for drug delivery and theranostics applications. In this review, we will summarize recent reports on the development of lipid and polymer based nanocarriers for targeted delivery of drugs and nucleic acids for the treatment of lung cancer.

#### 3.1.1 Drug Delivery

Drugs can be either encapsulated physically or bonded chemically through linker with the nanocarriers and can be delivered to almost all organs because of their small size and ease of penetration of many biological barriers. Over the years, a broad range of nanocarriers were evaluated for targeted delivery of several anticancer drugs for lung cancers. Few examples of lipid‐based nanoparticles as well as polymeric nanoparticles for targeted drug delivery applications have been summarized below.

##### Lipid‐Based Nanoparticles

Lipid‐based nanoparticles possess unique benefits necessary for drug delivery application. Lipid‐based nanoparticles have an advantage of being the least toxic among other nanocarriers with a substantial progress in the fields of drug and nucleic acid delivery using lipid‐based nanoassemblies.^[^ ^89^ ^]^ Here, we have summarized recent reports of several lipid‐based nanosystems including liposome, nanostructured lipid carriers (NLCs), and micelles and their application as targeted drug delivery systems.

**Figure 6:**
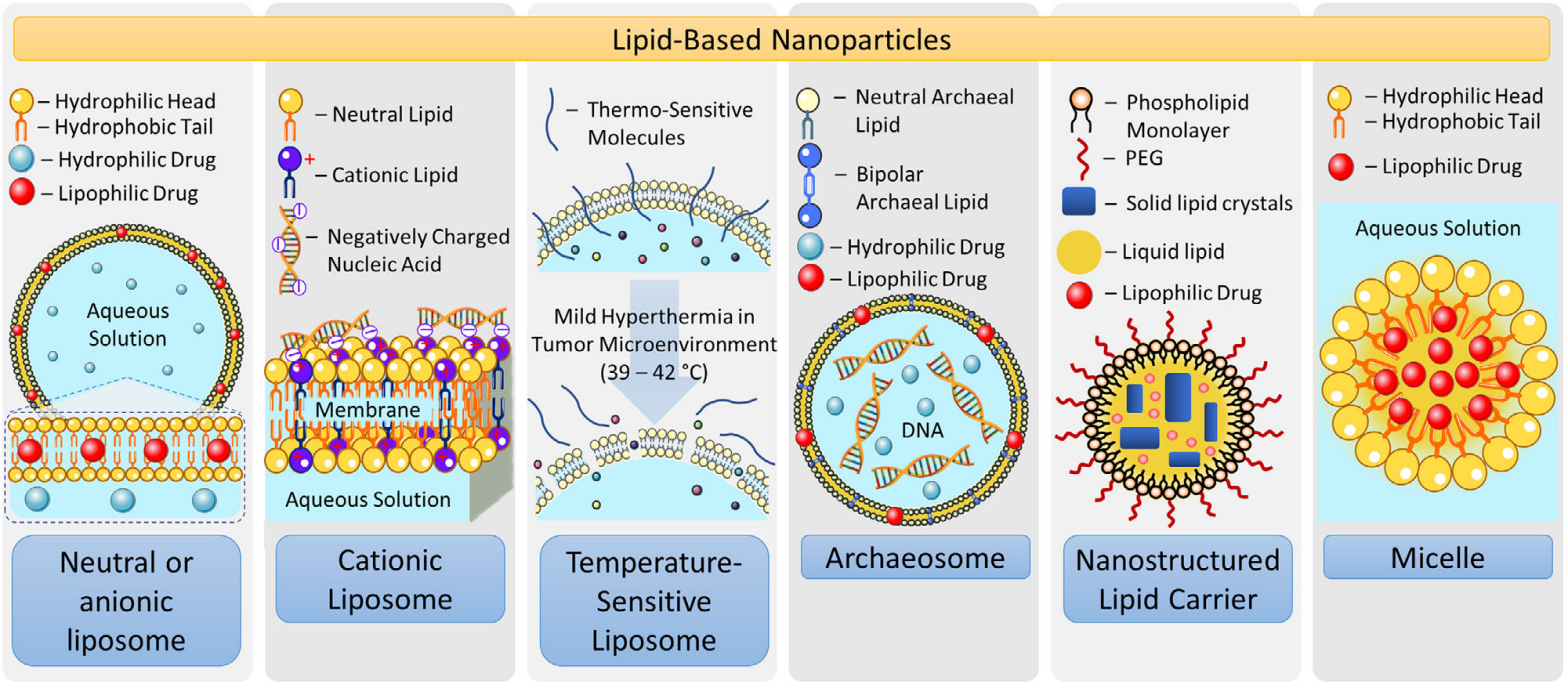
Examples of lipid‐based nanoscale‐based drug and nucleic acids delivery systems (not to scale). Typical structures of neutral, cationic and temperature‐sensitive liposomes, archaeosome (liposome made with one. or more ether lipids that are unique to the domain of Archaeobacteria), PEGylated nanostructured lipid carrier, and micelle with a single hydrocarbon chain in aqueous solution are shown.

*Liposomes*: Liposome is a type of lipid‐based nanoparticle with a bilayer structure comprised of phospholipids, phosphatidylcholine, cholesterol, etc. (**Figure** **6**). Liposome can incorporate lipid‐soluble drugs in its lipid bilayer structure as well as encapsulate water‐soluble drugs in its inner aqueous core.^[^ ^90^ ^]^ Liposomes were widely investigated for drug delivery applications because of their hydrophobic and hydrophilic drug loading capability as well as their biocompatibility properties.^[^ ^16^, ^23^, ^26^, ^27^, ^44^, ^91^, ^92^, ^93^, ^94^, ^95^ ^]^ Doxil is the first liposomal drug which got FDA approval as an anticancer nanotherapeutics in 1995.^[^ ^96^ ^]^ Over the last two decades, researchers have explored various liposomal formulations (such as temperature‐sensitive liposomes,^[^ ^97^ ^]^ cationic liposomes,^[^ ^98^ ^]^ and archaeosomes,^[^ ^99^ ^]^ etc.) for drug and gene delivery applications (Figure 6). For example, Song et al. developed a multifunctional liposome based complex system for in vivo treatment of NSCLC. The authors loaded an anticancer drug epirubicin inside the aqueous core of the liposome and an anti‐metastatic drug Honokiol into the lipid bilayer of the formulation. The surface of the liposome was further conjugated with a somatostatin targeting peptide octreotide which can binds to somatostatin receptors overexpressed in cancer microenvironment and facilitate targeted drug delivery. This complex liposomal system showed improved in vivo anticancer activity.^[^ ^100^ ^]^ Cisplatin is one of the widely used drugs for the treatment of lung cancer. However, it showed nephrotoxicity in patient with high doses.^[^ ^101^ ^]^ Devarajan et al. reported a liposomal formulation of cisplatin (namely Lipoplatin) which showed less nephrotoxicity when compared with free non‐bound cisplatin.^[^ ^102^ ^]^ Paclitaxel is another anticancer drug which was widely used for the treatment of various cancers including lung cancer. In a phase I clinical trial in NSCLC patients, a liposomal formulation of paclitaxel showed enhanced therapeutic efficacy.^[^ ^103^ ^]^ In a recent study, researchers prepared a liposomal formulation containing both cisplatin and paclitaxel for the treatment of lung cancer. In a phase III trial, this liposomal formulation displayed improved therapeutic activity and reduced nephrotoxicity in NSCLC patients.^[^ ^104^ ^]^ In the last decade, researchers have developed a special type of liposomes–archaeosomes, which are made with ether lipids unique to the domain of *Archaeobacteria*.^[^ ^105^ ^]^ Achaean‐type lipids consist of archaeol (diether) and/or caldarchaeol (tetraether) core structures. The membrane of this type of liposomes is made with both conventional phospholipids and bipolar lipids (Figure 6). Archaeosomes can be made using standard procedures used for liposome preparation including a sonication of hydrated film, extrusion, and detergent dialysis. In contrast to conventional liposomes, archaeosomes remain stable at acidic pH, high temperature and pressure, resist oxidative degradation and can be sterilized by autoclaving. A stability of archaeosomes at a wide physiological (or lower) temperature range opens a possibility of encapsulating thermally stable compounds. However, the uptake of archaeosomes by phagocytic cells can be up to 50‐fold greater when compared with conventional liposomes, which may be considered as their disadvantage.

*Nanostructured Lipid Carriers*: NLCs are another widely used lipid‐based nanocarrier for targeted drug and gene delivery applications. NLCs are composed of biodegradable and biocompatible lipids and usually prepared by mixing a liquid lipid mixture containing unsaturated lipid or oils to a solid lipid (Figure 6). Among the lipid‐based nanocarrier, NLCs have the advantages of ease of manufacturing processes, drug protection during the storage, low toxicity, biodegradation, etc. These and other benefits of NLCs made them a promising therapeutic delivery system in recent years.^[^ ^15^, ^25^, ^29^, ^106^ ^]^ Here, we have summarized few examples of NLC‐based therapeutic nanomedicines for drug and gene delivery. For instance, Guo et al.^[^ ^107^ ^]^ prepared a dual drug paclitaxel (PTX) and 5‐Demethylnobiletin (DMN) loaded and cetuximab (CET) functionalized CET‐PTX‐DMN‐NLCs for the combination therapy of lung cancer. The treatment of the CET‐PTX‐DMN‐NLC inhibited the growth of the lung cancer cells as compared to the single PTC‐NLC and DMN‐NLC treatments. The authors also observed remarkable inhibition of in vivo lung tumor for the treatment of this dual drug containing CET‐PTX‐DMN‐NLC.^[^ ^107^ ^]^ Wang et al.^[^ ^108^ ^]^ developed a dual drug loaded NLC system for the treatment of lung cancer. This dual drug loaded PTX/DOX NLC displayed 3× higher activity as compared to that of single drug PTC‐NLC and DOX‐NLC treatment as revealed in cytotoxicity assay in NCL‐H460 cells. Also, in vivo study of this dual drug NLC on a non‐small cell lung cancer mice model showed improved the anticancer activity.^[^ ^108^ ^]^

*Micelles*: Micelles consist of lipid molecules arranged in a spherical form in polar solvents (e.g., water). Polar groups of lipids form an outer shell of the nanoparticles in polar solvent system while lipid hydrophobic tails create an inner core of micelles. In contrast to liposomes, micelles usually have a single hydrocarbon chain (Figure 6). In 2007, Kim et al. prepared nanosized micelles (Genexol‐PM) loaded with the anticancer drug paclitaxel for the treatment of NSCLC.^[^ ^109^ ^]^ This nanotherapeutic was found to deliver higher paclitaxel dose with reduced drug toxicity as well as exhibited significant antitumor activity in the treatment of advanced NSCLC. A series of nanocarrier systems which included liposomes, micelles, quantum dots, mesoporous silica nanoparticles, dendrimers, and PEG polymers were prepared and examined in our laboratory in order to find out the suitable nanocarrier for local and inhalation delivery of anticancer drugs to the lungs. We investigated organ distribution and retention of all these nanocarriers in the lung and observed higher accumulation of liposomes and micelles based nanocarriers in lungs as compared to that of mesoporous silica nanoparticles, quantum dots, and dendrimers.^[^ ^26^ ^]^ We found a significant enhancement of anticancer activity of doxorubicin when it was delivered to mice bearing lung tumor by inhalation by liposome‐based system. This study revealed that lipid‐based nanocarriers such as liposomes with higher accumulation of drug in lungs and longer retention time were more suitable and effective than non‐lipid‐based nanocarriers in treating lung cancer by inhalation.^[^ ^26^ ^]^

##### Polymeric Nanoparticles

Polymeric nanoparticles are usually prepared by self‐assembly of various block‐copolymers with alternate hydrophobic unit between blocks. Various biodegradable polymers such as poly (lactic‐co‐glycolic) acid (PLGA), polycaprolactone, poly (lactic acid) (PLA), chitosan etc. were widely used for the preparation of polymeric nanoparticles mainly because of their biocompatibility and controlled release properties. While core‐shell of such polymeric nanoparticles can encapsulate hydrophobic drugs, the surface of the polymeric nanoparticles can be modified for receptor targeted drug delivery.^[^ ^110^, ^111^ ^]^ In the past two decades, many polymeric nanoparticles have been investigated for treatment of different diseases including lung cancers and enhancing efficacy of anticancer drugs.^[^ ^112^ ^]^

**Figure 7:**
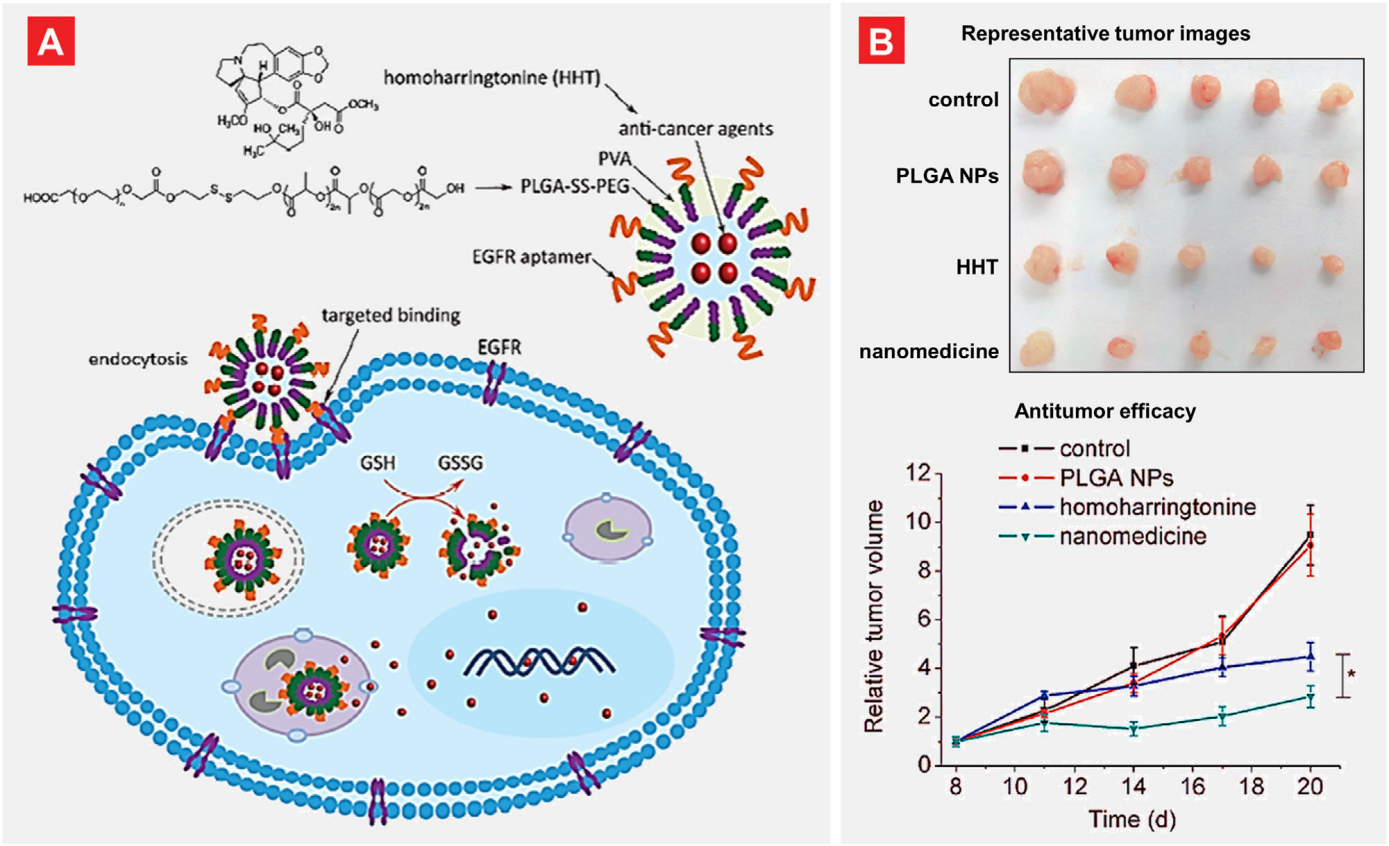
Glutathione stimuli responsive organic PLGA‐SS‐PEG nanocarriers for targeted delivery of anticancer agent homoharringtonine (HHT) to lung cancer cells. A) Core–shell structured nanoparticles were synthesized using the solvent evaporation approach of oil‐in‐water. The PLGA nanomedicine release was triggered by reduced glutathione (GSH) overexpressed in tumor cells after receptor mediated endocytosis. B) In vivo antitumor efficacy of PLGA nanomedicine. Treatment with PLGA‐SS‐PEG nanoparticles reduced the tumor volume significantly when compared with non‐treated and treated with free non‐bound HHT groups. Adapted with permission.^[^ ^48^ ^]^ Copyright 2017, Springer.

In a recent report, Hu et al.^[^ ^113^ ^]^ developed paclitaxel encapsulated polycaprolactone/poly (ethylene glycol)/polycaprolactone nanoparticles for the combined treatment of lung cancer with chronomodulated chemotherapy. This combination treatment showed better inhibition of tumor growth in vivo. Wang et al.^[^ ^114^ ^]^ developed a new strategy for delivering drug loaded polymeric nanoparticles at the disease site using mesenchymal stem cells (MSC) as carrier. Docetaxel was encapsulated in the nanoparticles and tested in vivo. Inhibition of the tumor growth was observed in the animal's experiment, which also revealed the translocation of nanoparticles from MSC to cancer cells. Researchers developed PEG modified and taxane encapsulated polylactic acid nanoparticles for lung cancer treatment. The authors observed significant improvement of the efficacy of chemoradiation therapy in an A549 lung tumor xenograft model.^[^ ^115^ ^]^ In another report, investigators developed a polymeric nanoparticle system comprised of block copolymers of PEG and polylactic acid and encapsulated paclitaxel and cisplatin for treatment of lung cancer.^[^ ^116^ ^]^ Tseng et al. prepared gelatin based polymeric nanoparticles conjugated with biotinylated EGF (bEGF) motif for EGFR‐targeted drug delivery.^[^ ^117^ ^]^ The authors observed enhanced cellular uptake of this polymeric nanoparticle in EGFR overexpressing cancer cell lines such as lung cancer cells. Zhang et al.^[^ ^118^ ^]^ studied glutathione stimuli responsive organic PLGA‐SS‐PEG nanocarriers for targeted delivery of anticancer agent homoharringtonine (HHT) to lung cancer cells. A relatively high level of glutathione reduced (GSH) in cancer cells caused disulfide‐bond (SS) breakage in a reductive manner and release of HHT inside cancer cells. This nanocarrier showed a reasonable biocompatibility and was further decorated with an epidermal growth factor receptor (EGFR) aptamer as a targeting moiety. After endocytosis of this nanosystem by lung cancer cells, the high level of glutathione in tumor cells stimulated the release of the loaded drug. Finally, this multifunctional and stimuli responsive nanocomplex inhibited the growth of human lung cancer cells and displayed better therapeutic efficacy when compared with the free non‐bound anticancer agent (**Figure** **7**).

##### Combined Lipid‐Polymer Nanoparticles

**Figure 8:**
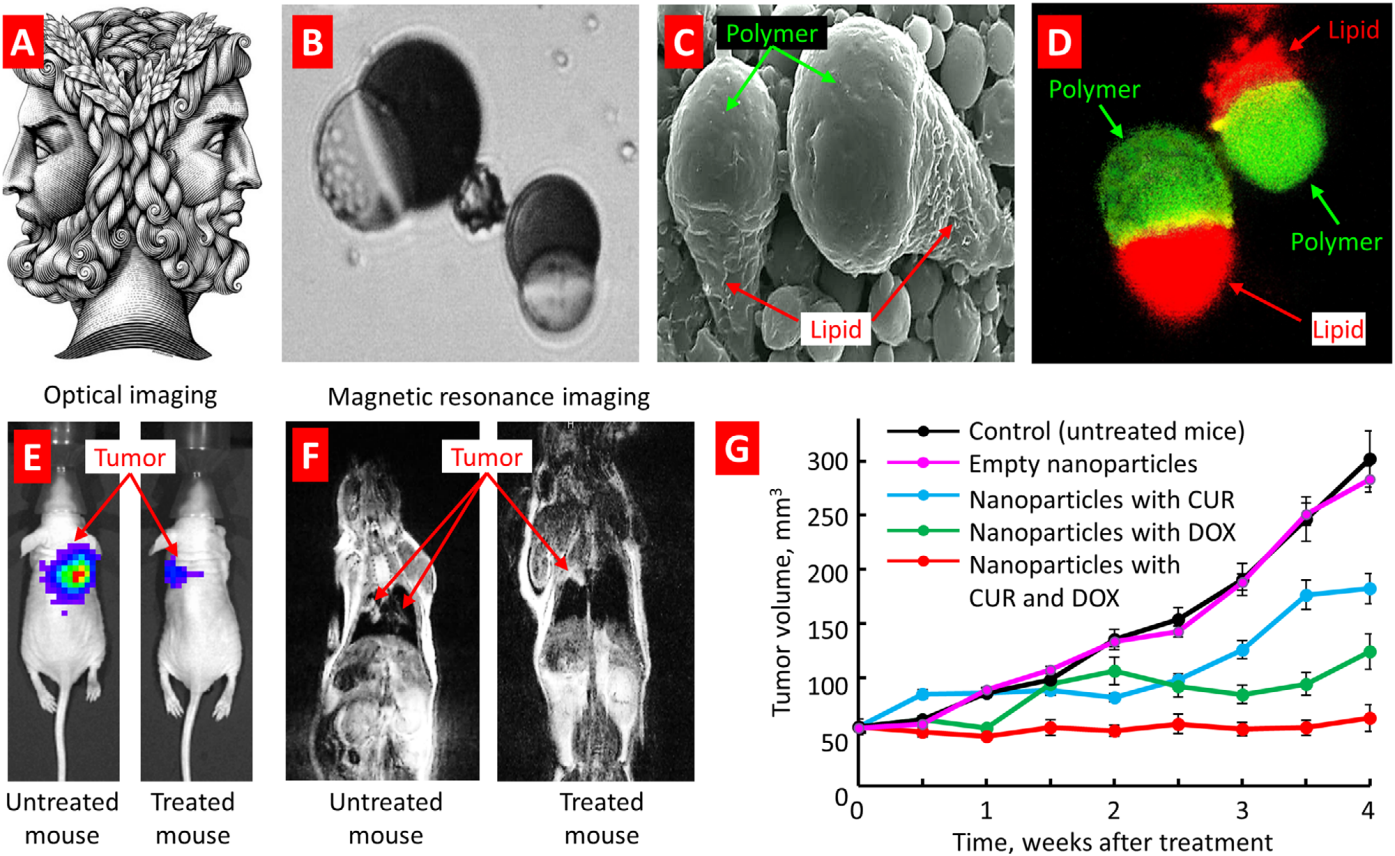
Suppression of lung tumor growth in mice treated by inhalation with Janus nanoparticles containing anticancer drug(s). A) Ancient Roman god of gates Janus who was portrayed with two faces (photo by A. Kokorin on Behance). B) Representative optical, C) scanning electron and D) fluorescence microscope images of anisotropic biodegradable biphasic polymer/lipid Janus nanoparticles. Polymeric phase of nanoparticles was labeled with FITC (green fluorescence); lipid phase was labelled with DiR (red fluorescence). E) Representative optical and F) magnetic resonance images of untreated (control) and treated mice four weeks after tumor instillation. G) Changes in lung tumor volume after beginning of the treatment with nanoparticles containing doxorubicin (DOX), curcumin (CUR), and both drugs. Mice were treated twice per week. Means ± SD are shown. Reproduced with permission.^[^ ^28^ ^]^ Copyright 2014, ACS Publications.

In addition of nanocarriers prepared mainly with lipids or polymers, complex anisotropic nanoparticles containing lipid and polymeric structures (as well as polymers with different properties) were prepared.^[^ ^119^, ^120^, ^121^, ^122^, ^123^, ^124^ ^]^ As Pierre‐Gilles de Gennes pointed in his 1991 Nobel Prize lecture,^[^ ^125^ ^]^ similarly to the ancient Roman god of gates Janus who was portrayed with two faces—one facing the past, and one facing the future, Janus particles also have two distinct parts with antagonistic properties (**Figure** **8**). One such Janus structure with two faces (lipid and polymeric) was tested in our laboratory for inhalation lung delivery of a mixture of lipo‐ and hydrophilic drugs namely curcumin and doxorubicin.^[^ ^28^ ^]^ These Janus particles were synthesized from binary mixture of biodegradable and biocompatible materials and evaluated for cytotoxicity and genotoxicity. The inhibition of lung tumor growth by the combination treatment was significantly higher when compared with either free drugs or nanoparticles containing only one drug. This study showed that such Janus particle could be explored for the simultaneous co‐delivery of hydrophilic and hydrophobic drugs.

##### Carbon Nanomaterials

Carbon nanomaterials are a new class of nanosized materials comprised of sp2 hybridized carbon atoms with hexagonal structure. Common carbon nanomaterials include 0D fullerenes, 1D carbon nanotubes (CNTs), and 2D graphene such as graphene oxide.^[^ ^126^ ^]^ Among the carbon nanomaterials, CNTs have received significant attention in the past two decades for their high surface area, biocompatibility and drug‐loading capacity which made them suitable for wide range of applications such as drug delivery, tissue engineering, biosensors, cosmetic products etc.^[^ ^127^, ^128^ ^]^. There are mainly three types CNTs namely single wall‐, double wall‐ and multi wall‐ carbon nanotubes with diameter up to 100 nm and lengths up to microns size.^[^ ^129^, ^130^ ^]^ Surface of these CNTs can be functionalized with hydrophobic and hydrophilic drugs for their targeted delivery applications. Carbon nanomaterials such as CNTs were explored as an attractive systems in targeted drug delivery application. In 2017, Kim et al. developed PEG‐coated carbon nanotube system loaded with small molecule BCL‐2 inhibitor ABT‐737 for its targeted delivery to lung cancer cells.^[^ ^131^ ^]^ The authors investigated cellular uptake, apoptosis, and cytotoxicity this PEG‐CNT‐ABT737 nanotube system in lung cancer A549 cells and observed BCL‐2‐mediated apoptosis of lung cancer cells. The PEG‐CNT‐ABT737 system also excreted improved cytotoxic activity in A549 cells when compared with treatment by free non‐bound ABT737. The drug loaded nanotubes represented an effective system for inducing BCL‐2‐mediated apoptosis in lung cancer cells. In 2019, Cirillo et al. reported a pH‐responsive nanohybrid system comprised of multi‐walled carbon nanotubes and chitosan for delivery of methotrexate to lung cancer cells.^[^ ^132^ ^]^ This chitosan coated CS‐MWCNT nanohybrid system displayed its pH‐responsive behavior and showed faster and higher release of the drug methotrexate in acidic (pH 5.0) versus neutral (pH 7.4) environments. Such a nanohybrid system showed reduced drug toxicity in normal lung MRC‐5 cells while it exerted anticancer activity in lung cancer H1299 cells.

##### Mesoporous Silica Nanoparticles

Nanosized silica particles also known as mesoporous silica nanoparticles (MSNs) have been investigated in the past two decades for various drug and gene delivery application. Because of large pore size, high surface area, good chemical stability, biocompatibility, and ease of surface modification with targeting ligands, MSNs based nanomaterials were extensively studied for various therapeutic delivery applications.^[^ ^6^, ^30^, ^131^, ^133^, ^134^ ^]^ For example, Wang et al. designed a nanosized drug delivery system containing anticancer drug paclitaxel into the core‐shell of mesoporous silica nanoparticle (PAC‐csMSN) for the treatment of lung cancer.^[^ ^135^ ^]^ This csMSNs formulation improved the adsorption of the poorly water‐soluble drug paclitaxel. The authors found PAC‐csMSN system was more effective in promoting cell apoptosis in A549 lung cancer cells than the free drug. This PAC‐csMSN system was administered for three consecutive days in animals and no indication of inflammation was observed in the lung biopsy. All these results indicated that such PAC‐csMSN system has the potential for inhalation delivery of paclitaxel for the treatment of lung cancer. 2018, Jing‐Hua Sun et al. prepared another MSNs system for co‐delivery of a photosensitizer chlorin e6 (Ce6) and a drug doxorubicin (Dox) for both photodynamic therapy and chemotherapy of lung cancer.^[^ ^136^ ^]^ The anticancer drug doxorubicin was encapsulated into the pores of MSNs system while Ce6 was conjugated with the MSNs through covalent bonding. Treatment with these Dox@MSNs‐Ce6 hybrid nanoparticles increased the level of cellular reactive oxygen species and exerted synergistic therapeutic effect in lung cancer A549 cells when compared with treatment by each individual component.

##### Gold Nanoparticles

Gold nanoparticles (AuNPs) are one of the extensively used inorganic nanocarriers for various biomedical applications including drug and gene delivery. Because of high atomic number, stable nature and surface plasmon resonance properties, AuNPs can serve as stable contrast agents for photothermal therapy and medical imaging.^[^ ^137^, ^138^ ^]^ Moreover, AuNPs possess unique physiochemical characteristics such as high biocompatibility and low‐toxicity as well as AuNPs are non‐immunogenic which made gold nanoparticles as an attractive nanocarriers for various biomedical applications.^[^ ^139^, ^140^, ^141^ ^]^ In 2014, Qian et al. conjugated Cetuximab (C225), a targeting agent for epidermal growth factor receptor (EGFR) with AuNPs for the treatment of EGFR positive non‐small cell lung cancer (NSCLC).^[^ ^142^ ^]^ This C225‐AuNPs inhibited proliferation and migration of A549 cells and accelerated apoptosis in A549 cells as compared to treatment with free C225 alone. The activity of C225‐AuNPs was higher in A549 cells with higher EGFR expression than in H1299 cells with low EGFR expression. Treatment of nude mice bearing tumor xenografts with C225‐AuNPs showed significant suppression of tumor size. Such EGFR‐targeted AuNPs system can be a promising strategy for targeted delivery of therapeutics in EGFR positive NSCLC cells. In 2018, Ramalingam et al. conjugated doxorubicin on the surface of gold nanoparticles through polyvinylpyrrolidone linker for the treatment of human lung cancer cells.^[^ ^143^ ^]^ These Dox‐PVP‐Au nanoparticles inhibited the growth of human lung cancer cells more effectively than both the PVP‐AuNPs and free drug. Treatment with these nanoparticles induced early and late apoptosis as well as upregulated expression of tumor suppressor genes in the human lung cancer cells. This Dox‐PVP‐Au nanoparticle system represents a promising drug delivery approach for lung cancer therapy.

##### Cell Based Drug Carriers

**Figure 9:**
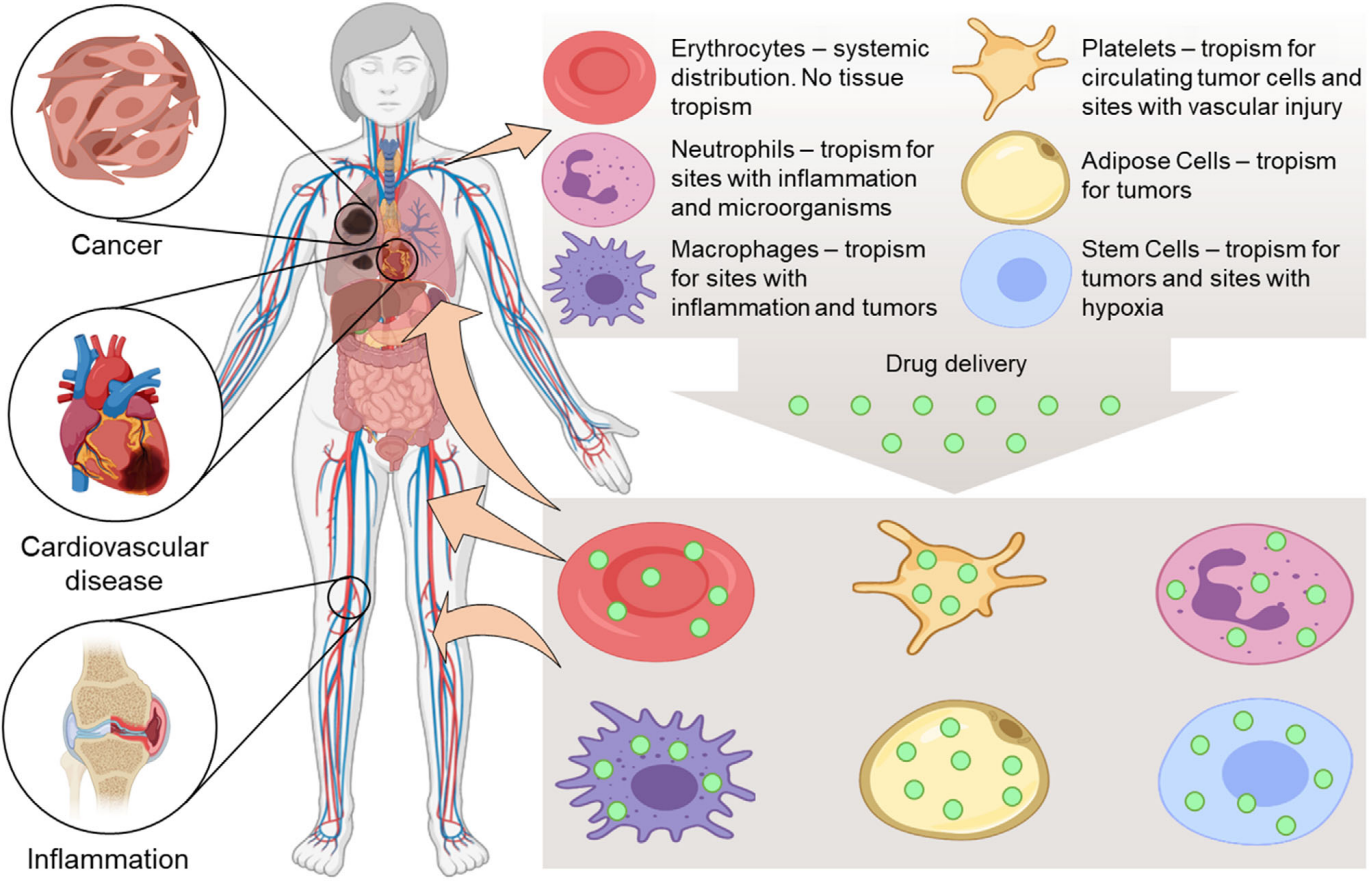
Various cell types employed for drug delivery. Redrawn from.^[^ ^144^, ^145^ ^]^

Biocompatibility, biodegradability, and cytotoxicity of synthetic nanocarriers represent a substantial problem. Moreover, exogenous carriers potentially may induce immune responses. Consequently, drug carriers prepared from human live cells or their derivatives attract a considerable attention in recent years. Such carriers demonstrate native targeting mechanisms and controlled release of the encapsulated drug molecules. Several types of human cells have been considered for a targeted drug delivery for treatment of cancers and variety of other pathological conditions, such as cardiovascular and inflammatory diseases.^[^ ^144^, ^145^ ^]^ Major characteristics of different cell types used for drug delivery are presenting in the **Figure** **9**.

Erythrocytes are usually used for a systemic drug delivery and do not possess intrinsic tropism. In contrast, platelets, neutrophils, adipose cells, macrophages, and stem cells can be used for targeted delivery of therapeutics because of their native tropism to tumors, circulating tumor cells, sites with inflammation, and hypoxic conditions as well as microorganisms.^[^ ^145^ ^]^ A strategy of drug loading into human cells used for therapeutic delivery is selected based on the nature and properties of drugs, loading capacity and release mechanisms. Therapeutic agents can be loaded into cellular cytoplasm of carrier cells, attached to their surface by a membrane insertion, nonspecific noncovalent or targeted interaction as well as covalent coupling methods. The release of payload may be continuous (e.g., after slow hydrolysis of a prodrug and exocytosis) or triggered by various internal in vivo signals (glucose, hormones, cytokines, and other biomolecules, pH, and changes in cell shape) or external stimuli (light, ultrasound, magnetic field, temperature, etc.).^[^ ^145^ ^]^ A comprehensive list of cell based carriers designed for intraperitoneal, intratracheal, intravenous, subcutaneous and left anterior descending delivery of therapeutics developed during the last decade is presented in the open access review by Lutz et al.^[^ ^144^ ^]^

#### 3.1.2 Delivery of Nucleic Acids

**Figure 10:**
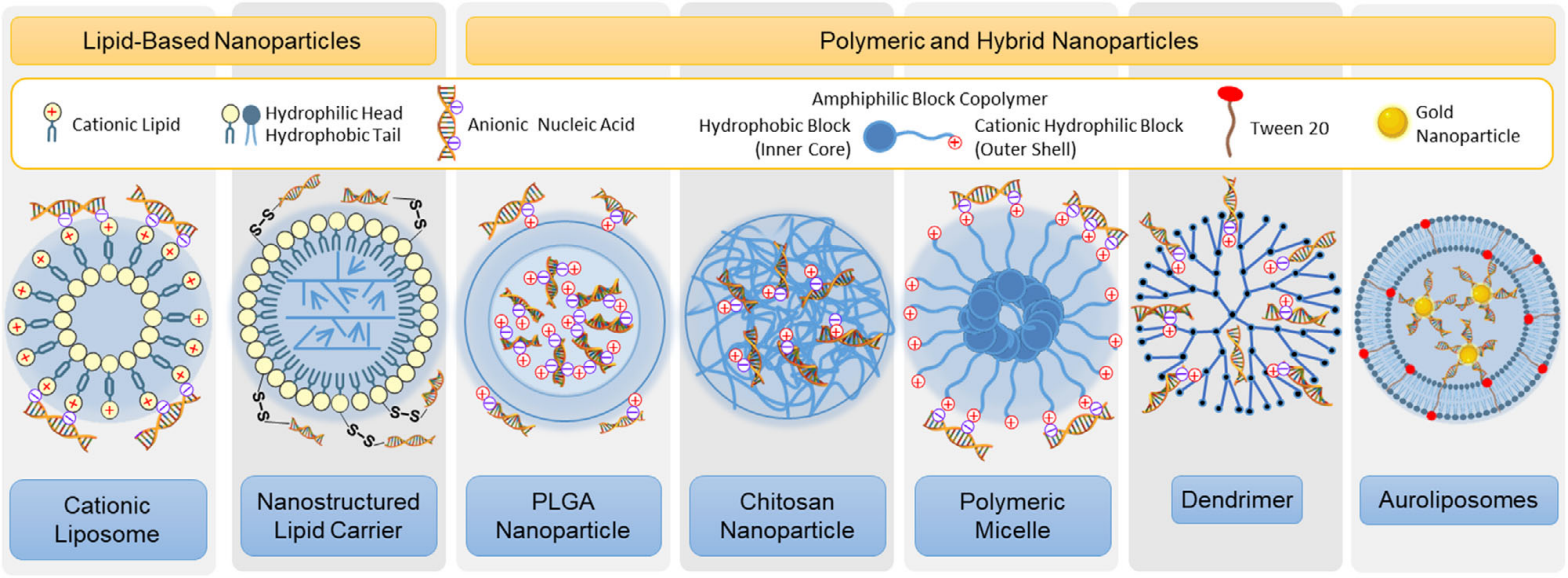
Commonly used nanoparticles for the delivery of nucleic acids (not to scale). Negatively charged nucleic acids are usually integrated with nanoparticles by electrostatic interactions or chemical conjugation. Depending on the size of nucleic acid, it may be fully or partially entrapped inside one or several nanoparticles or bound to the surface of nanoparticle(s).

Gene therapy has become a promising therapeutic option in recent time for lung cancer. Several different nanocarriers (such as dendrimer, micelles, gold nanoparticles, liposomes, lipid nanoparticles, auroliposome etc.) were explored as carriers of nucleic acids with effective results.^[^ ^14^, ^15^, ^23^, ^25^, ^27^, ^29^, ^42^, ^43^, ^45^, ^92^, ^134^, ^146^, ^147^, ^148^, ^149^ ^]^ Examples of carriers used for the delivery of nucleic acids are presented in **Figure** **10**. Nucleic acids used as gene therapeutics are negatively charged because they are composed of few, several or many nucleotides with phosphate backbones carried one negative charge per residue.^[^ ^150^ ^]^ Consequently, they often delivered as conjugates formed with positively charged (cationic) carriers. Such conjugation not only protects gene material from degradation in the blood stream and improves pharmacokinetics of the resulting complex, but also neutralizes positive charge of highly toxic anionic carriers limiting their cyto‐ and genotoxicity.^[^ ^151^ ^]^ However, an encapsulation inside nanocarriers or direct conjugation of native or modified nucleic acids via different (preferably cleaved inside targeted cells, e.g. S─S) bonds are also used to form a stable system for an effective gene delivery. Small chunks of nucleic acids can be modified to decrease their negative charge and encapsulated inside nanocarriers. In our laboratory, the DNA backbone of all bases in antisense oligonucleotides (ASO) was P‐ethoxy modified in order to make the entire ASO neutral and increase their incorporation efficacy into liposomes.^[^ ^149^ ^]^ Such modification also enhanced nuclease resistance of ASO. Liposomal ASO were successfully used to suppress pump and nonpump resistance of cancer cells.^[^ ^27^, ^41^, ^43^, ^91^ ^]^ It should be stressed, that treatment of cancer with nucleic acids alone (e.g., siRNA, antisense oligonucleotides) in most cases demonstrate a pretty limited anticancer effect. However, a combination of anticancer drug(s) with nucleic acids targeted to the drug efflux pumps, antiapoptotic, and other cancer cell defensive proteins/mRNAs is expected to substantially enhance anticancer efficacy of both anticancer drugs and nucleic acids. Such a concept of advanced proapoptotic anticancer delivery system was first developed and tested in the laboratory of Professor Minko at Rutgers University almost 20 years ago.^[^ ^36^, ^37^ ^]^ Possible structures of such multifunctional nanoparticles are presented in Figure 1. In several such systems, where nucleic acids (siRNA and antisense oligonucleotides) were used as suppressors of drug efflux pumps (pump drug resistance) and antiapoptotic cellular defense (non‐pump resistance).^[^ ^54^, ^91^ ^]^

##### Lipid‐Based Nanoparticles

Lipid‐based nanoparticles such as liposomes, NLCs, and various polymeric nanoparticles are widely used to protect and deliver nucleic acids (Figure 10). Few examples of both liposome and NLC based nucleic acid delivery systems are discussed below.

##### Liposomes

Gopalan et al. prepared DOTAP/cholesterol based nanocarrier system for direct delivery of tumor suppressive gene at the tumor site. This system was effective and non‐immunogenic.^[^ ^152^ ^]^ In an early study, our group prepared a multicomponent liposomal delivery system for improving anticancer activity of doxorubicin against multidrug‐resistant human non‐small‐cell lung cancer cells. This multi‐component liposomal system was included doxorubicin as an anticancer drug, antisense oligonucleotide (ASO) as a suppressor of pump resistance for MRP1 mRNA and another ASO as a suppressor of nonpump resistance for BCL2 mRNA.^[^ ^27^, ^41^, ^43^, ^92^, ^149^ ^]^ Antisense oligonucleotides were P‐ethoxy modified to decrease their charge, enhance nuclease resistance, and increase incorporation efficacy into liposomes. We reported successful intracellular delivery of both doxorubicin and ASOs to lung cancer cells. Also, this liposomal treatment increased anticancer efficacy of doxorubicin and inhibited synthesis of both MRP1 and BCL2 proteins. This multicomponent liposomal system displayed 10‐fold higher cytotoxicity as compared to both free and liposomal doxorubicin treatment against the resistant lung cancer cells and could be used for the enhancement of anticancer activity of doxorubicin against multidrug‐resistant lung cancer cells. In a similar study, we used a complex liposomal drug delivery system containing anticancer drug doxorubicin and both MRP1 and BCL2 targeting antisense oligonucleotides for inhalation treatment in lung cancer cells.^[^ ^27^ ^]^ While empty liposome, free antisense oligonucleotides and their combination treatment showed almost no influence on viability of lung cancer cells; liposome targeted to both MRP1 mRNA and BCL2 mRNA significantly inhibited the growth of the lung cancer cells. We evaluated this complex liposomal system on an orthotopic murine model of human lung cancer and the results revealed its higher chemotherapeutic efficacy with lower side effects as compared to that observed for individual treatment of each component.^[^ ^27^ ^]^

##### Nanostructured Lipid Arriers

Garbuzenko et al. reported a multi‐functional nanostructured lipid carrier (LHRH‐NLC‐siRNAs‐TAX) composed of an anticancer drug paclitaxel (TAX), a peptide analog targeted to luteinizing hormone‐releasing hormone (LHRH) receptor and a pool of siRNAs as inhibitors of different types of EGFR‐TKs. This LHRH‐NLC‐siRNAs‐TAX nanoparticle system was investigated in various human lung cancer cell lines and in vivo on an orthotopic NSCLC mouse model and displayed good organ distribution, stability, solubility, and improved anticancer activity when compared with free individual drugs and non‐targeted therapy.^[^ ^15^, ^25^, ^29^ ^]^

Han et al. developed a multifunctional BLC system for delivery of anticancer drug doxorubicin (DOX) and green fluorescent protein plasmid (pEGFP) DNA as a prototypical nucleic acid in lung cancer cells. The authors prepared DOX and pEGFP encapsulated NLC and modified its surface with transferrin‐targeting motif. Transferrin‐modified and DOX and pEGFP co‐encapsulated NLC system showed higher in vitro and in vivo transfection of plasmid DNA than the other control treatment. These results also indicated that such multifunctional NLC system could be an effective method for both drug and gene delivery for the treatment of lung cancer.^[^ ^153^ ^]^ In another work, Han et al. prepared an NLC system for delivery of plasmid‐containing enhanced green fluorescence protein (pEGFP) in lung cancer cells. The authors prepared the pEGFP‐loaded NLC and decorated its surface with transferrin (Tf) targeting ligands. This Tf‐NLC/pEGFP showed higher transfection efficiency as observed in in vitro and in vivo studies than the non‐targeted NLC/pEGFP–suggesting this NLC system could be a promising vehicle for gene therapy in lung cancer.^[^ ^154^ ^]^

##### Polymeric and Hybrid Nanoparticles

Chitosan‐based polymeric nanoparticles were widely used for the delivery of nucleic acids. Because of ionizable sidechain amino groups in chitosan, it has cationic nature which made chitosan polymer a good vehicle for the delivery of anionic agents such as siRNA, DNA etc. Okamoto et al. developed a chitosan‐based nanoparticle for delivery of pCMV‐Luc gene into the lung cancer cells.^[^ ^155^ ^]^ Nafee et al. prepared a chitosan based nanocarrier system loaded with an antisense oligonucleotide such as 2‐*O*‐Methyl‐RNA for the treatment of lung cancer. The authors evaluated the inhibitory function of telomerase after treating the nanocarrier in lung cancer cells and observed 50% reduction of the telomerase activity in A549 lung cancer cells. These chitosan nanoparticles were safe and effective for the treatment of lung cancer.^[^ ^156^ ^]^ Dhananjay et al. developed a polymeric nanoparticle system comprised of PEI‐PEG copolymer for the delivery of Akt1 shRNA in lung cancer cells.^[^ ^157^ ^]^ Both service and internally anionic polypropylenimine tetrahexacontaamine (PPI) and polyamidoamine (PAMAM) dendrimers were successfully used for the delivery of nucleic acids into cancer cells.^[^ ^158^, ^159^, ^160^ ^]^ It was found that dendrimers and additional caging of resulting nanoplexes protected nucleic acids from degradation and effectively delivered genetic material inside cancer cells. Delivered siRNA demonstrated high intracellular activity and effectively knocked down gene expression and synthesis of targeted proteins.

##### Carbon Nanomaterials

Carbon nanomaterials such as CNTs decorated with positively charged polymers were investigated as gene carrier in recent years for specific delivery of nucleic acids.^[^ ^161^, ^162^ ^]^ Podesta et al. prepared amino‐functionalized MWCNT system for delivery of siRNA and tested it using an animal xenograft and orthotopic breast tumor models.^[^ ^137^ ^]^ Treatment with this MWNCT‐siRNA complex significantly delayed the growth of tumor and increased the survival of the tumor bearing animals. Varkouhi et al. developed MWCNT systems decorated with cationic PEI (CNT‐PEI) and CNT–pyridinium for the delivery of siRNA to lung cancer cells.^[^ ^163^ ^]^ Both these CNTs displayed cytotoxicity effect and gene silencing activity in H1299 human lung cancer cells, while non functionalized CNTs did not show any such effects.

##### Mesoporous Silica Nanoparticles

Mesoporous silica nanoparticles have been investigated as a delivery vehicle of various cargo molecules including nucleic acid therapeutics. For example, Dilnawaz et al. developed MSN based system for co‐delivery of anticancer drug doxorubicin and siRNA in lung cancer cells.^[^ ^164^ ^]^ This combinational treatment enhanced in vitro cellular uptake, cytotoxic effect in A549 lung cancer cells. In 2020, Song Yinxue et al. developed a complex MSN system comprised of a polyphenolic drug Myricetin (Myr), siRNA specific to multidrug resistance protein (MRP‐1) and a targeting ligand folic acid (FA) in order to improve delivery efficiency of Myr in NSCLC cells.^[^ ^165^ ^]^ This targeted Myr‐MRP‐1/MSN‐FA nanoparticles showed significant cellular uptake and reduced viability of A549, NCI‐H1299 lung cancer cells when compared with free drug and other controls. In vivo results revealed that this system was more effective in suppressing the tumor growth and it might be an attractive therapeutic strategy for the treatment of NSCLC.

##### Gold Nanoparticles

Gold nanoparticles possess good biodistribution, physiological stability and low cytotoxicity which made them an attractive vehicle for delivery of various payloads including large biomolecules such as nucleic acids.^[^ ^166^ ^]^ Over the years, researchers have explored both non‐covalent and covalent conjugation of the nucleic acids such as siRNA, oligonucleotides etc. on the surface of AuNPs for their effective transportation to the target cells. For instances, Conde et al. developed a PEG modified gold nanoparticle system by conjugating of RGD peptide and c‐myc siRNA on the surface of gold nanoparticles and tested this system on mice bearing CMT/167 lung carcinoma tumors.^[^ ^167^ ^]^ The authors observed downregulation of the c‐myc oncogene and significant inhibition of lung tumor growth after the treatment with si‐RNA/RGD AuNPs. Recently, an innovative hybrid formulation (so‐called auroliposomes) consisting of liposomes loaded with 20‐nm gold nanoparticles (AuNPs) was developed and used for the siRNA delivery (Figure 10). It was found that auroliposomes modulated the intracellular uptake and silencing efficacy leading to the enhanced suppression of tumor growth in vivo when compared with conventional liposomes.^[^ ^168^ ^]^

#### 3.1.3 Diagnostics and Theranostics

Nanocarriers have the potential to enhance the diagnosis of diseases. Recently, nanobased materials and methods have emerged as novel diagnostic tools for several diseases. Over the years, various nanocarriers were explored for the delivery of imaging (or both imaging and therapeutic) agents for diagnosis of many diseases including lung cancer.^[^ ^169^, ^170^, ^171^ ^]^ For example, researchers designed folic acid functionalized dendrimers containing gold nanocarrier as cancer‐targeted imaging probes for computed tomography (CT) imaging of lung cancer cells.^[^ ^172^ ^]^ CT imaging after nanoparticle uptake revealed the presence of these gold nanocarriers in the lysosomes of lung adenocarcinoma cells. In another study, researchers developed ultra‐small (3.0 ± 0.1 nm) Gadolinium containing nanoparticles (so called ultra‐small rigid platforms or USRPs) for enhancing Ultrashort Echo Time (276 ms) proton MRI of the lung.^[^ ^173^ ^]^ These nanoparticles were prepared using 1,4,7,10‐tetraazacyclo‐dodecane‐1,4,7,10‐tetraacetic acid (DOTA) as a chelator and was delivered by the intratracheal instillation. The authors observed the substantial (>250%) enhancement of MRI signal in the lungs for almost 2.5 h after instilling the solution of the nanoparticles.

Erten et al. prepared a dextran core‐based stealth PEGylated liposomes containing anticancer drug doxorubicin, iron oxide as an MRI contrast agent and Boron dipyrromethene (BODIPY) fluorescence stain for imaging theranostics applications.^[^ ^174^ ^]^ The authors observed strong ability of these liposomal nanoparticles of enhancing both types of imaging in the in vivo murine model of Lewis lung cancer. In another report, Lowery et al. labeled a tumor targeted doxorubicin loaded liposomes with Alexa Fluor 750 for imaging of lung tumor.^[^ ^175^ ^]^ An HVGGSSV peptide with a selective binding to irradiated tumors was used as a targeting moiety in order to deliver the anticancer drug and imaging agent specifically to irradiated tumors limiting their accumulation in the normal tissues. These liposomes (100 nm) contained maleimide and amine functionalized PEG chains for the conjugation of the cysteine containing peptide and the *N*‐(Succinyl)‐fluorophore, respectively. Doxorubicin in theranostic liposomes was loaded by the pH gradient. The authors studied these fluorophore labeled irradiated tumor targeted liposomes in murine model of Lewis lung cancer. They found that such a radiation‐guided tumor‐targeted delivery of liposomes enhanced the delivery of the fluorophore and anticancer drug specifically to irradiated tumors in the lungs, effectively induced cell death and limited cell proliferation within lung tumors finally inducing a delay in tumor growth and destruction of tumor blood vessels and increase of apoptosis in lung tumor cells. HVGGSSV targeting peptide also increase the accumulation of an entire system in irradiated tumors enhancing imaging quality.

### 3.2 Idiopathic Pulmonary and Cystic Fibrosis

Cystic fibrosis is an inherited disorder, caused by mutations of the cystic fibrosis transmembrane conductance regulator (CFTR) gene. Over‐production of mucous in the lungs causes airway obstruction resulting in infectious diseases such as cystic fibrosis (CF).^[^ ^176^ ^]^ Typically, heterogeneous and large molecular weight oligomeric gel‐forming mucin glycoprotein are produced in CF. Gene therapy is the mainstay therapy to inhibit the mutation of CFTR protein. Gene therapies involve delivery of siRNA, DNA etc. into cells to rescue the function of the defective CFTR gene. Usually, viral and non‐viral vectors are employed to transfer correct copies of CFTR gene in the effected cells in lungs.^[^ ^177^ ^]^ Because of small size, nanocarriers have emerged as an effective vehicle for delivery of gene through the mucus barriers. Nanoscale carriers can be used as vectors for gene therapy due to their less immunogenic and good gene transport capacity.^[^ ^178^ ^]^ Nanocarrier based non‐viral vectors are easy to prepare as compared to that of viral vectors.^[^ ^179^ ^]^ In recent years, researchers attempted to develop various nucleic acid based nanocarrier to affect mutation of CFTR gene to change the composition of mucin as well as to minimize the mucin production.^[^ ^180^, ^181^ ^]^

In an early research, Konstan et al. developed a DNA nanoparticle for the treatment of cystic fibrosis and observed effective transfer of vector gene.^[^ ^182^ ^]^ In order to overcome the mucus barrier, recently, Suk et al. prepared a densely PEG‐coated DNA nanoparticle system, which can penetrate extracorporeal human cystic fibrosis to deliver its payload.^[^ ^183^ ^]^ This nanocarrier displayed better gene transfer after intranasal administration to mice as compared to other carriers. Minko et al. prepared a liposomal‐α‐tocopherol (LAT) formulation for the treatment of hypoxic lung injury in rats. The authors evaluated antioxidant and antiapoptotic activity of this LAT in rats with severe hypoxia and observed significant antihypoxic effects.^[^ ^93^ ^]^ It was found that, treatment with LAT of rats under severe hypoxic conditions (breathing of 6% of oxygen within two hours) normalized lung phospholipid composition, inhibited lipid peroxidation, suppressed genes responsible for the development of lung damage and improved breathing pattern. Finally, such a treatment two‐times decreased the mortality of the animals under severe hypoxic conditions. Lately in our lab, a similar liposomal system for inhalation delivery of prostaglandin E2 (PGE2) was developed for treatment of pulmonary fibrosis.^[^ ^16^ ^]^ This liposomal system was evaluated for local delivery of PGE2 using a standard bleomycin‐induced murine model of idiopathic pulmonary fibrosis. The results revealed that liposomes were accumulated in higher amount in lungs after inhalation delivery when compared with intravenous administration. Besides, this inhalation treatment reduced fibrotic injury in the lung tissues. These data probed that the inhalation administration of liposomal form of PGE2 can be an effective therapy for cystic fibrosis in the lungs. To further improve inhalation treatment of idiopathic pulmonary fibrosis (IPF) by liposomal PGE2, siRNAs targeted to major proteins responsible for the lung damage under IPF (MMP3, CCL12, and HIF1A) were added to the NLC based nanoparticles containing PGE2 and tested on the similar experimental model of lung fibrosis using inhalation delivery.^[^ ^23^ ^]^ This enhanced advance system was more effective in the treatment of IPF when compared with siRNA and PGE2 delivered separately. Another combination of drugs in one NLC‐based nanoparticle system was recently tested for the treatment of lung manifestation of cystic fibrosis (CF).^[^ ^24^ ^]^ The system included lumacaftor for the correction of correct p.Phe508del mutation (the loss of phenylalanine at position 508) and CFTR potentiator ivacaftor for increasing the open probability of CFTR channels. This system was tested in vitro using CF cells and in vivo on homozygote/homozygote bi‐transgenic mice with spontaneously developed CF. The system was delivered in vivo by inhalation. The results showed a high efficacy of the proposed treatment of the lung manifestation of CF. Wang et al. prepared rapamycin and azithromycin loaded polymeric nanocarrier via nanoprecipitation method.^[^ ^184^ ^]^ Nanocomposite microparticles (nCmP) were formulated from this nanoparticle for the inhalation delivery of antibiotics in the form of dry powder aerosols. These, nanocomposite microparticles displayed aerosol dispersion characteristics indicating their deposit in the lungs.

### 3.3 Coronavirus Diseases

Viral infections in respiratory systems such as in lungs have become a worldwide public health threat in recent years. Several emerging positive‐stranded RNA coronaviruses^[^ ^185^, ^186^ ^]^ such as Severe Acute Respiratory Syndrome Coronavirus (SARS‐CoV),^[^ ^187^, ^188^, ^189^ ^]^ Middle East Respiratory Syndrome Coronavirus (MERS‐CoV)^[^ ^191^ ^]^ etc. not only threatened public health, but also caused international epidemics in the past two decades. Recent outbreak of coronavirus infection caused by the severe acute respiratory syndrome‐coronavirus‐2 pathogen has seriously threatened public health all over the world. The taxonomic name “severe acute respiratory syndrome coronavirus 2” (SARS‐CoV‐2) given by the International Committee on Taxonomy of Viruses (ICTV) became official to refer to this virus strain. On February 11, 2020 the World Health Organization (WHO) officially named the “coronavirus disease 2019” as “COVID‐19”.^[^ ^192^ ^]^ The genome of SARS‐CoV‐2 is a 29 903 bp with single‐stranded RNA (ss‐RNA). The complete genome sequence of SARS‐CoV‐2 is available in the National Center for Biotechnology1 (NCBI) database, with ID NC_04 5512.^[^ ^193^, ^194^ ^]^ COVID‐19 is characterized by severe respiratory disease along with mild to high fever, cough, and shortness of breath. COVID‐19 has been considered as an emerging disease and on March 11, 2020 the outbreak of this disease has been declared as global pandemic by the WHO.^[^ ^195^, ^196^ ^]^ This virus has been found to spread from person to person mainly through respiratory droplets, cough, sneeze, etc.^[^ ^197^ ^]^ causing severe acute respiratory distress syndrome (ARDS). As of September 20, 2020, this virus has already infected more than thirty million people and caused 950000 deaths with billions of people are at risk around the world.^[^ ^198^ ^]^ Despite repeated outbreaks of SARS‐CoV in 2003 and MERS‐CoV in 2012, no potent vaccines and anti‐viral drugs are commercially available against these viral infections—mainly due to the fact that the outbreaks of these viruses were rapidly contained and did not reappear.^[^ ^199^ ^]^ Therefore, there are no effective treatment for the ongoing pandemic of COVID‐19, a close subtype of SARS‐CoV.^[^ ^200^ ^]^ Because of constant emergence of new viruses including current SARS‐CoV‐2 infection, there is an urgent need for the development of potent and broad‐spectrum vaccines and antiviral drugs for effective control of viral diseases. Since the first report of SARS‐CoV‐2 infection in late December in 2019, both researchers and clinicians have been attempted clinical trials of several known antiviral drugs, their combination as well as development of vaccine in patients with confirmed COVID‐19 disease. This review is mainly focused for summarizing recent developments of nanotherapeutics for respiratory diseases including SARS‐CoV, MERS‐CoV, and COVID‐19. Therefore, other types of therapeutic and diagnostic methods such as small molecule antiviral therapeutics, anti‐SARS‐CoV‐2 antibody treatments, convalescent plasma therapy etc.^[^ ^201^, ^202^, ^203^, ^204^ ^]^ which have been discussed elsewhere are out of the scope of this review. Briefly, we will summarize recent innovation of nanobased diagnostics such as nanoparticle‐based PCR and anti‐body test as well as nanoparticle‐based therapeutic approaches for COVID‐19.

#### 3.3.1 Diagnostic Approaches

**Figure 11:**
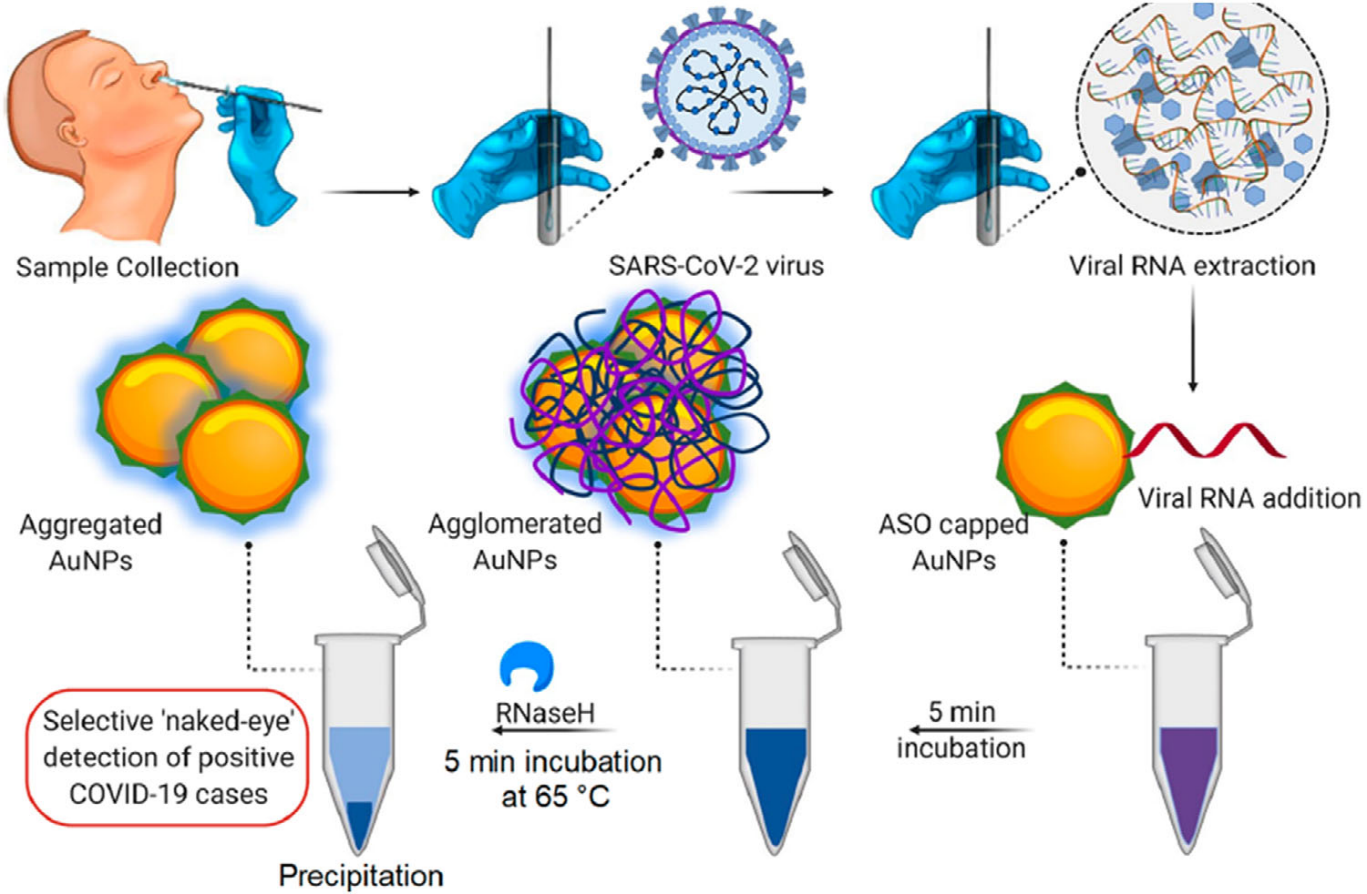
Schematic representation for the selective naked‐eye detection of SARS‐CoV‐2 RNA mediated by the suitably designed ASO‐capped gold nanoparticles. This naked eye detection method involves the isolation of the viral RNA from the clinical swab sample from COVID‐19 patient and then incubation of the viral RNA samples with ASO capped gold nanoparticles for 5 min. At the end, RNase H is added in the viral composite of ASO‐capped gold nanoparticles and the resulting mixture is incubated for 5 min at 65 C to get the visual precipitate. Reproduced with permission.^[^ ^208^ ^]^ Copyright 2020, ACS Publications.

Diagnostic tests are essential not only for monitoring every stage of a disease, but also to identify new patients with that illness–especially for an outbreak of viral disease. Typical diagnosis methods for viral diseases include nucleic acid detection of the viral genome in clinical samples. Currently, COVID‐19 has been diagnosed by real‐time polymerase chain reaction (RT‐PCR) test for the detection of viral genome, serological, and immunological assays for the detection of anti‐SARS‐CoV‐2 antibody in patient samples as well as chest computed tomography (CT) imaging for screening abnormal observations in chest scans.^[^ ^205^, ^206^ ^]^ However, most of these methods are laborious and time‐consuming processes. Therefore, there is an urgent need for developing time‐economic, easily performed and point‐of‐contact test for the detection of this virus. Because of similar size and shape of SARS‐CoV‐2 virus with the synthetic nanoparticles, researchers have attempted to develop nanoparticle based diagnostic methods for COVID‐19. For examples, Huang et al. developed a rapid, easily operated and cost‐effective detection of the IgM antibody produced in serum sample of patient with COVID‐19.^[^ ^207^ ^]^ The authors prepared a colloidal gold nanoparticle‐based lateral‐flow (AuNP‐LF) system composed of various low‐cost inorganic nanomaterials. The AuNP‐LF strip was developed for the sample test by coating an analytical membrane with the SARS‐CoV‐2 nucleoprotein followed by conjugating anti‐human IgM antibody. This method can detect the SARS‐CoV‐2 virus in 15 min using only 20 µL of serum sample of the patient. The authors have evaluated the specificity of this detection method against the results of widely used TR‐PCR's test. This AuNP‐LF assay has a great potential for large‐scale and fast detection of COVID‐19 disease specially during this pandemic period.^[^ ^207^ ^]^ In another report, Moitra et al. developed gold nanoparticle based colorimetric assay for naked‐eye detection of SARS‐CoV‐2 virus present in patient samples.^[^ ^208^ ^]^ The authors decorated the gold nanoparticles (AuNPs) with thiol‐modified antisense oligonucleotides (ASOs) which are specific for N‐gene (nucleocapsid phosphoprotein) of SARS‐CoV‐2 virus. The use of RNaseH in this detection helps to cleave the RNA–DNA hybrid resulting in the visually detectable precipitation from the experimental solution. This AuNP system can detect the presence of SARS‐CoV‐2 stain in the isolated RNA samples within 10 min. Thus, this method can be a very promising for visual detection of COVID‐19 positive patient without the use of typical instrumental procedures as outlined in **Figure** **11**.^[^ ^208^ ^]^

#### 3.3.2 Therapeutic Approaches

**Figure 12:**
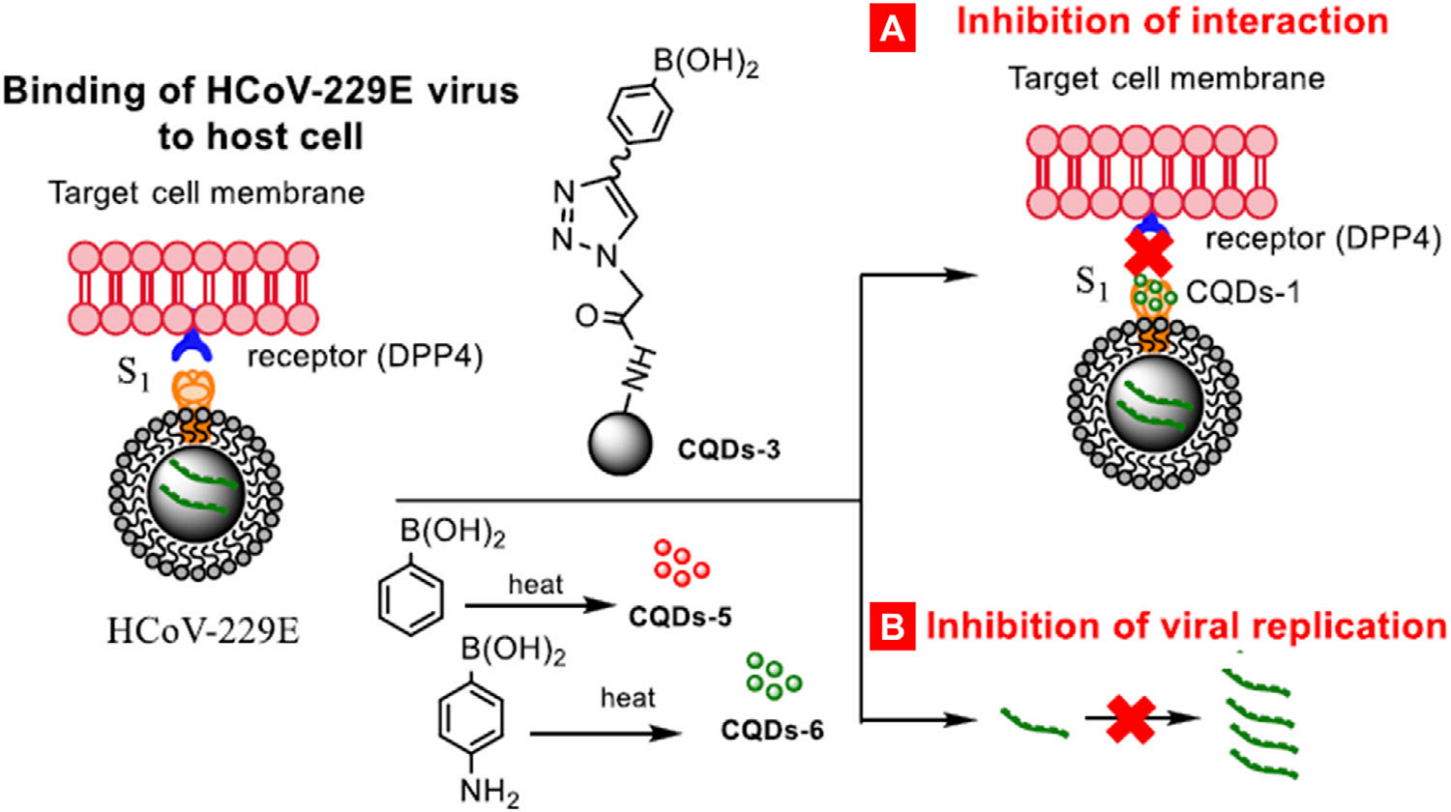
Influence of carbon quantum dots, prepared by hydrothermal carbonization, on binding of HCoV‐229E virus to cells: A) inhibition of protein S receptor interaction, and B) inhibition of viral RNA genome replication. Boronic acid adducts based carbon quantum dots (CQDs) derived from phenylboronic acid and 4‐aminophenylboronic acid showed antiviral activity mainly by inhibiting the entry of HCoV‐229E virus as well as inhibiting the replication step of the viral genome. Reproduced with permission.^[^ ^217^ ^]^ Copyright 2019, ACS Publications.

There are no clinically approved therapeutics by the U.S. Food and Drug Administration (FDA) to prevent or treat the COVID‐19 disease. However, clinical trials of many known anti‐viral drugs are ongoing in patient with confirmed COVID‐19 disease.^[^ ^209^, ^210^ ^]^ For instance, a newly developed antiviral drug Remdesivir previously effective against Ebola virus diseases which interferes with viral RNA polymerase (RdRp) and arrests viral replication^[^ ^211^ ^]^ was repurposed for the treatment of COVID‐19 infections.^[^ ^212^ ^]^ Human trials showed promising clinical improvements in ≈70% of patients.^[^ ^213^ ^]^ Recent studies showed that the SARS‐CoV‐2 virus has similar size range (50–150 nm) and spherical shape like the synthetic nanoparticles.^[^ ^214^ ^]^ Therefore, nanosized spherical therapeutics can be promising to detect and neutralize coronaviruses as previously evaluated for SARS‐CoV, MERS‐CoV etc. Various nanoparticle based therapeutic approaches have been discussed here for MERS‐CoV, SARS‐CoV, and COVID‐19 diseases. For instance, Huang et al. prepared gold nanorod complex of heptad repeat 1 (HR1) peptide inhibitors for Middle East respiratory syndrome coronavirus (MERS‐CoV) disease.^[^ ^215^ ^]^ This gold nanorod complex was biocompatible and metabolically stable and displayed 10‐fold higher inhibition of membrane fusion between host cells and MERS‐CoV via HR1/HR2‐mechanism as compared to that of the free inhibitor treatment. This the gold nanorod based anti‐viral system showed a great promise in treating MERS‐CoV infection. Lin et al. prepared a virus‐like hollow nanoparticle comprised of biodegradable polymer and a viral antigen along with an adjuvant.^[^ ^216^ ^]^ This plasmid like nanoparticle was capable of delivering of both antigens and stimulator of interferon genes agonist adjuvant to induce potentiation to the immune cells. The authors observed that this nanoparticle‐based MERS‐CoV vaccine was effective against a lethal dose of MERS‐CoV infection as compared to control treatment in a MERS‐CoV‐permissive transgenic mouse model. The potency of this nanoparticle‐based vaccine for MERS‐CoV was demonstrated, and this study provides a new outline for developing nanocarrier based vaccine for viral pathogen. Loczechin et al. prepared seven different carbon quantum dots (CQDs) and investigated their anti‐viral activity against the human coronavirus HCoV‐229E infections.^[^ ^217^ ^]^ These CQDs displayed a concentration‐dependent virus inactivation and CQD produced from 4‐aminophenylboronic acid showed better activity with EC_50_ 5.2 ± 0.7 µg·mL^−1^. The mechanistic studies revealed that interaction of the surface functional groups of the CQDs with entry receptors of the HCoV‐229E virus resulted in inhibition of the infection. These results suggested such CQDs systems might be explored for developing anti‐viral therapeutics for other coronavirus infections (**Figure** **12**).^[^ ^217^ ^]^ Polymeric nanoparticles consisting of poly (ethylene glycol)‐block‐poly(lactide‐coglycolide) (PEG‐PLGA) were developed for delivery of diphyllin, a novel vacuolar ATPase blocker for its antiviral activity against the feline coronavirus infection.^[^ ^218^ ^]^ Treatment with these nanoparticles significantly reduced toxicity and enhanced antiviral effect of diphyllin. These nanoparticles were well tolerated as revealed in animal study in mice following high‐dose intravenous administration. The results of the study indicated that such diphyllin nanoparticles could be explored as effective host‐targeted antiviral therapeutics for other coronavirus infections. Coleman et al. developed a novel strategy for preparing spike nanoparticles which in combination with adjuvants produced high titer anti‐bodies in mice against both the severe acute respiratory syndrome coronavirus (SARS‐CoV) and Middle East Respiratory Syndrome Coronavirus (MERS‐CoV) infections.^[^ ^219^ ^]^ The results showed that these spike nanoparticles were able to neutralize the antibody responses in mice—suggesting a step towards nanovaccine development.^[^ ^219^ ^]^ Recently, fabric material‐based face mask containing hydrophilic absorbent layers and hydrophobic barrier layers was constructed.^[^ ^220^ ^]^ This fabric masks were found to show equivalent or better filtration and adsorption of nanoparticle like aerosols than the commercial N95 respirators. The aerosols were composed of fluorescent labeled virus like nanoparticles for tracking their transmission through the fabric masks. The authors evaluated 70 different combinations of common fabric materials using forced convection air flux with pulsed aerosols. This fabric masks can be used to protect from the inhalation of such viruses. In an early study, Li et. al reported that treatment based on RNA interference (RNAi) exhibited antiviral immunity in mammals.^[^ ^221^ ^]^ In 2018, Sohrab S. et al. designed and developed a series of lipid and polymeric nanoparticles for delivery of therapeutic siRNA for the treatment of MERS‐CoV infection.^[^ ^222^ ^]^ Since SARS‐CoV‐2 is a single strand RNA virus like other coronaviruses, therefore inhibiting the life cycle of SARS‐CoV‐2 viruses via silencing their viral mRNA in the host cells by RNA interference might be an effective therapy for COVID‐19. Recently, both researchers and clinicians have been working in developing nanoparticle encapsulated mRNA‐based vaccines for COVID‐19. For examples, vaccine candidate mRNA‐1273 (Moderna) is a lipid nanocarrier based mRNA vaccine that encodes spike protein of SARS‐CoV‐2 virus. In an early study (clinical trial identifier: NCT04283461), Jackson et al. conducted a phase I open label clinical trial of the mRNA‐1273 in 45 adults with 15 people in each group of 25, 100, and 250 µg dose.^[^ ^223^ ^]^ Each group of people received second vaccination after 28 days of the first vaccination. Systematic side effects such as headache, fatigue, pain at the injection site etc. were observed in half the participants particularly after second vaccination with higher dose treatment. Initial results revealed that treatment of mRNA‐1273 induced immune responses in all participants and the antibody responses were higher for the higher dose treatment participants. Recently, Corbett et al. studied this mRNA‐1273 vaccine in nonhuman primates and observed that treatment with this vaccine candidate increased neutralizing antibody levels that were higher than in human convalescent‐phase serum sample.^[^ ^223^ ^]^ The mRNA‐1273 vaccine is now under further evaluation for COVID‐19. BNT162b1 is another nanoparticle encapsulated mRNA vaccine candidate that encodes receptor binding domain (RBD) of spike glycoprotein of SARS‐CoV‐2. Recently, Mulligan et al. conducted a placebo‐controlled Phase 1/2 clinical trial of nucleoside‐modified mRNA vaccine which was formulated in a lipid‐based nanoparticle system for targeting RBD of spike glycoprotein of SARS‐CoV‐2 virus (clinical trial identifier: NCT04368728).^[^ ^223^ ^]^ The authors performed a randomized and placebo‐controlled trial of BNT162b1 vaccine in 45 healthy adults. There were 12 participants for each of the dose level 10, 30, and 100 µg of the vaccination and nine participants in placebo with BNT162b1 increased the SARS‐CoV‐2 neutralizing titers with dose level in the serum sample. Thus, nanosized therapeutics such as nanoformulation of anti‐viral drugs, nanovaccines, etc. can be promising options for the diagnosis and treatment of such viral infection.

## 4 Conclusions and Outlook

As summarized in this review, the past two decades have witnessed substantial amount of work in the development and applications of nanocarrier based systems for targeted delivery of drugs, gene, imaging agents etc. as well as nanoparticle‐based diagnostics for various respiratory diseases. Several preclinical and clinical investigations revealed that nanocarrier‐based systems address many limitations of conventional therapy not only by site‐specific delivery of therapeutics at the lung tissue, but also reducing the drug availability into other organs thereby reducing adverse side effects. Besides, nanocarrier based systems demonstrated sustain and control release of the therapeutics than the burst release observed in systematic delivery of therapeutics in lungs. Moreover, nanosized carriers have a potential to overcome the mucus barrier and poor lung penetration associated with various respiratory diseases. Similarly, recent development of various nanoparticle‐based detection methods for coronavirus infection showed a great promise in the development of time‐economic diagnosis of COVID‐19 disease. While such nanoscale systems promise new therapeutic options for respiratory diseases, still the most challenging task is their safety assessment. Preparation of an appropriate size of nanoparticles in each batch of synthesis is also challenging. Many such challenges need to be overcome in order to translate the nanotherapeutics into clinical practice. Though nanotechnology can find a way for further application of nanotherapeutics against the COVID‐19 diseases, still more research needs to be conducted for evaluation of nanosized therapeutics for COVID‐19. It has been considered that the effective vaccine against COVID‐19 will be available in 12–18 months. Therefore, early diagnosis, effective treatment etc. are essentials to mitigate the spread of this infection before any clinically approved vaccine comes in the market. Finally, the following should be mentioned. Because the major result of COVID‐19 infections is acute respiratory distress syndrome, the discussed nanomedicines for treatment lung hypoxia and fibrosis potentially can be used for the treatment of COVID‐19 in combination with other antiviral actions. Such pilot investigations have been recently initiated in our laboratory.

## Conflict of Interest

The authors declare no conflict of interest.
